# Molecular Modeling of Isoniazid Encapsulation by β-Cyclodextrin for Drug Delivery: Molecular Dynamics, Interaction Energies, and DFT Insights

**DOI:** 10.3390/ijms27219838

**Published:** 2026-11-03

**Authors:** Al-shimaa S. M. Rady, Peter A. Sidhom, Shaista Zarrin Khanam, Mohamed K. Abd El-Rahman, Lamiaa A. Mohamed, Shaban R. M. Sayed, Tamer Shoeib, Mahmoud A. A. Ibrahim

**Affiliations:** 1 Computational Chemistry Laboratory, Chemistry Department, Faculty of Science, Minia University, Minia 61519, Egypt; 2 Department of Pharmaceutical Chemistry, Faculty of Pharmacy, Tanta University, Tanta 31527, Egypt; 3 Department of Supportive Requirements, University of Technology and Applied Sciences, Nizwa 611, Oman; 4 Department of Chemistry and Chemical Biology, Harvard University, 12 Oxford Street, Cambridge, MA 02138, USA; 5 Central Laboratory, College of Science, King Saud University, P.O. Box 2455, Riyadh 11451, Saudi Arabia; 6 Department of Biological, Chemical and Global Health Sciences, School of Sciences and Engineering, The American University in Cairo, New Cairo 11835, Egypt; 7 School of Health Sciences, University of KwaZulu-Natal, Westville Campus, Durban 4000, South Africa

**Keywords:** β-Cyclodextrin, isoniazid drug, host–guest complexation, MD simulation, DFT calculations, quantum theory of atoms in molecules

## Abstract

β-cyclodextrin (β-CD) plays a crucial role in advanced drug delivery processes, with potential applications in drug protection and release modulation. Therefore, the encapsulation potential of β-CD towards isoniazid (ISO) was herein comprehensively examined by performing molecular dynamics (MD) simulation with density functional theory (DFT) calculations. From an energetic perspective, the encapsulation process within the ISO⋯β-CD complex was energetically preferable, as evidenced by the negative interaction energy (*E*_int_) values of configurations A–E. Compared to the other configurations, configuration A exhibited the most favorable *E*_int_, reaching −41.66 kcal/mol. From FMO and TDOS analyses, a reduction in energy gap values was observed following the encapsulation process, indicating a change in the electronic structure of the ISO⋯β-CD complex. The effect of water on the encapsulation process within the ISO⋯β-CD complex was investigated. Furthermore, the potential release of ISO from β-CD was qualitatively assessed using simplified recovery-time estimates. Moreover, ISO adsorption over the β-CD surface was examined using the same approaches as the encapsulation model; however, it was energetically less favorable, with *E*_int_ values reaching only −18.42 kcal/mol. Overall, the MD-enhanced DFT results underscored the potential of β-CD as a candidate platform for ISO drug encapsulation; however, experimental validation is required to establish loading efficiency, release behavior, bioavailability, and toxicity.

## 1. Introduction

Nanomaterials with promising properties have been substantially utilized across various disciplines and have led to notable advancements [1,2,3,4]. In biological applications, nanocarriers with biocompatible and bioavailable features have been widely used as drug delivery systems and biosensors [5,6,7]. Nanocarriers have been widely employed as an innovative strategy to address the poor water solubility and toxicity limitations of drugs [8,9,10,11]. Subsequently, supramolecular nanocarriers have been extensively applied to various biomedical aspects [12,13].

Cyclodextrins (CDs), first isolated by Villiers in 1891, are starch-derived cyclic α-(1 → 4)-linked glucooligosaccharides and possess a hydrophilic exterior and a relatively less polar cavity [14,15,16]. In aqueous solution, the cavity accommodates water molecules that may be displaced upon inclusion of suitable guest species. The most common native CDs are α-CD, β-CD, and γ-CD, which consist of six, seven, and eight α-D-glucopyranose units, respectively [17]. However, larger-ring cyclodextrin homologs and other families of cyclic oligosaccharides have also been identified [18,19]. Owing to their truncated-cone structures, CDs can encapsulate drugs, which in turn enhances the solubility and stability of the drug [20]. Among CDs, β-CD, which has a suitable cavity size, has emerged as a versatile supramolecular carrier that improves drug solubility, stability, and bioavailability while enabling targeted and controlled release [21,22,23,24]. Indeed, β-CD’s potential for delivering drugs was theoretically and experimentally examined [25,26,27,28,29].

Isoniazid (ISO), an FDA-approved drug, has been used since 1952 as a cornerstone of tuberculosis treatment [30,31]. ISO, with the chemical formula C_6_H_7_N_3_O, exhibits strong antibacterial activity and is highly recommended as a first-line treatment for tuberculosis [32,33,34]. Nevertheless, ISO causes several side effects, including hepatotoxicity and peripheral neuropathy [35]. Previous studies have investigated nanoclusters, heterofullerenes, and quantum dots as drug delivery systems for ISO [36,37,38]. ISO complexation with cyclodextrins has also been examined using calorimetry, ^1^H NMR spectroscopy, volumetric measurements, and molecular docking [39,40,41,42].

Despite the high aqueous solubility of ISO, computational investigations of its complexation with β-CD provide molecular-level insights into host–guest interactions and a preliminary basis for exploring the potential benefits of cyclodextrin-based drug delivery systems, particularly protection against degradation and the modulation of drug release. Nevertheless, these potential pharmaceutical advantages, including improved drug stability, controlled release, and reduced adverse effects, require further experimental validation. Accordingly, the present study aimed to investigate the structural stability, energetic favorability, and noncovalent interactions of ISO encapsulation within β-CD using density functional theory (DFT) calculations and molecular dynamics (MD) simulation. MD simulation was first performed for the encapsulated ISO⋯β-CD complex, followed by clustering analysis of the collected MD snapshots. Consequently, various DFT computations were performed on the MD-derived snapshots, including geometry optimization, electrostatic potential (ESP) analysis, and energy calculations. Furthermore, noncovalent interaction (NCI), symmetry-adapted perturbation theory (SAPT), and frontier molecular orbital (FMO) analyses were executed. In addition, total density of states (TDOS) and quantum theory of atoms in molecules (QTAIM) analyses were executed. As an essential consideration, the water impact on the stability of the ISO⋯β-CD complex was examined. Moreover, the ISO adsorption process over the β-CD surface was subsequently investigated using the same approaches as the encapsulation model. The current study provides an in-depth evaluation of the drug encapsulation potential of β-CD, particularly for the ISO drug.

## 2. Results and Discussion

### 2.1. ESP Analysis

**Figure 1:**
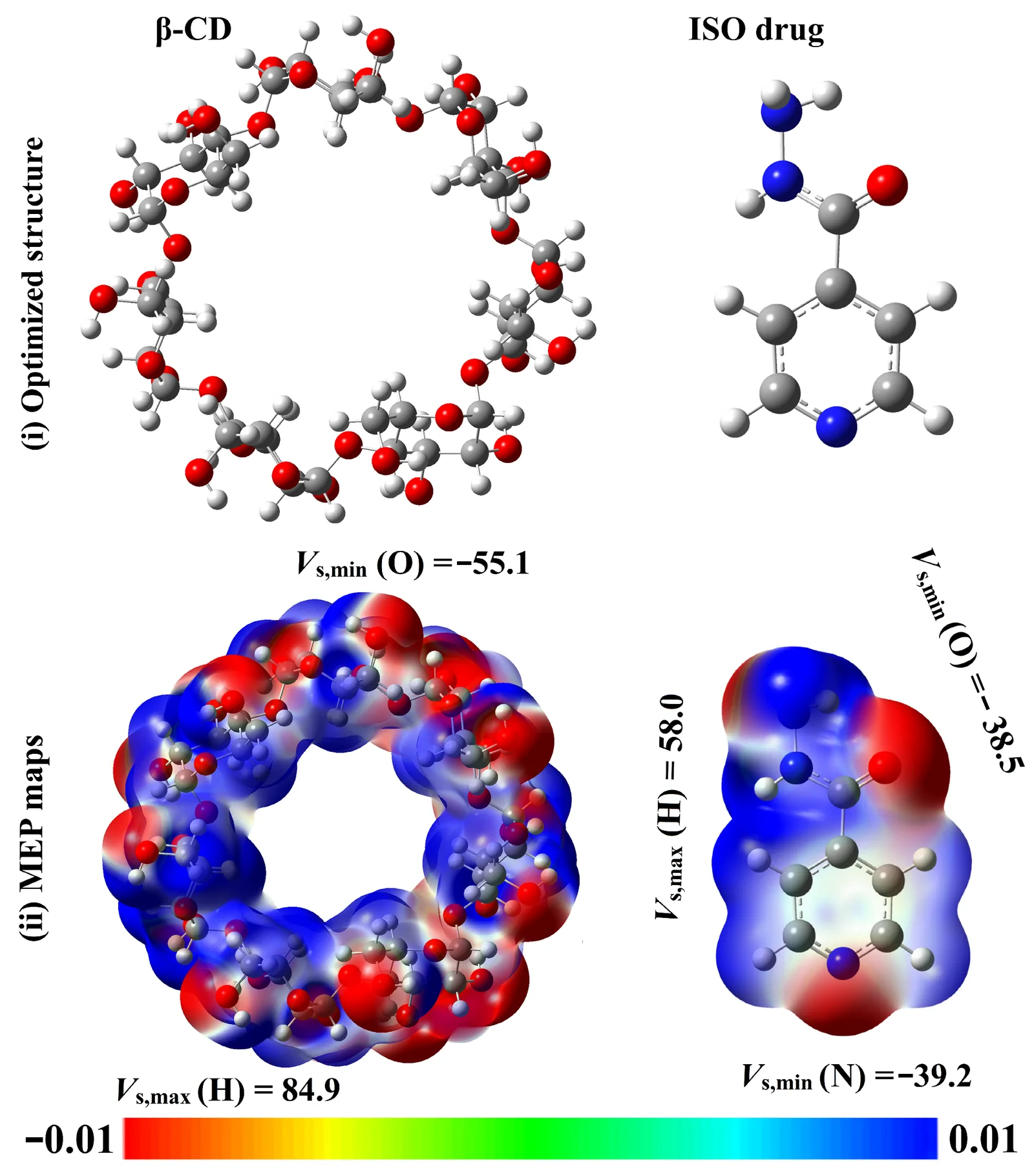
(**i**) Optimized structures and (**ii**) MEP maps supported by *V*_s,min_/*V*_s,max_ values (in kcal/mol) for β-CD and ISO. MEP color scale ranged from +0.01 au (blue) to −0.01 au (red).

ESP analysis offers meaningful insights into the distribution of electrostatic potential around a molecular structure [43,44]. Accordingly, MEP maps of β-CD and ISO were generated and *V*_s,min_/*V*_s,max_ values were estimated (Figure 1).

Figure 1 illustrates that the optimized β-CD structure is formed by seven D-glucopyranose units containing C, O, and H atoms, organized in a truncated-cone structure. The related MEP maps displayed regions of negative ESP (red) around the O atoms and positive ESP (blue) over portions of the H/C-containing regions. As numerical evidence, the red and blue regions were supplemented by *V*_s,min_ and *V*_s,max_ values reaching −55.1 and 84.9 kcal/mol, respectively.

In the MEP maps of ISO, the N and O atoms were colored red, exhibiting *V*_s,min_ values reaching −39.2 and −38.5 kcal/mol, respectively. Meanwhile, H atoms were colored blue, with *V*_s,max_ values up to 58.0 kcal/mol. Collectively, the ESP analysis suggested pronounced interaction tendencies between ISO and β-CD, supporting the feasibility of ISO encapsulation into β-CD.

### 2.2. Molecular Dynamics (MD) Simulation

To gain insights into the encapsulation behavior, the preassembled ISO···β-CD complex was subjected to a 10 ns MD simulation to assess its structural stability and dynamic behavior over the simulated timescale. For the generated MD snapshots, clustering analysis was carried out, yielding several clusters of the ISO⋯β-CD complex. Consequently, the lowest-energy conformation from each cluster was selected as a representative configuration and subsequently subjected to DFT computations. This approach characterized the conformational behavior of the preassembled complex but did not demonstrate convergence of conformational sampling, spontaneous encapsulation, or equilibrium binding.

### 2.3. Encapsulation Process

**Figure 2:**
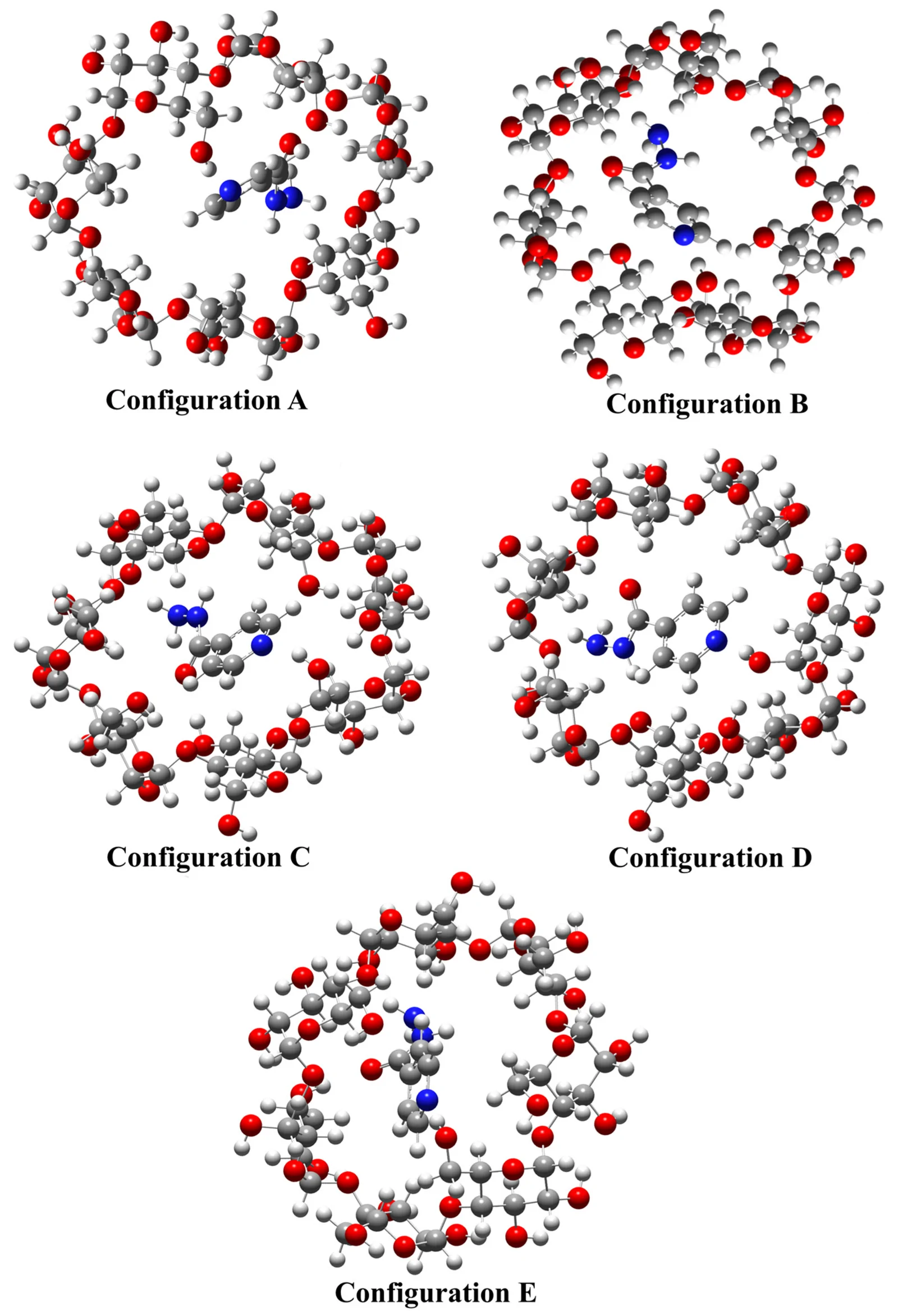
Top views of the optimized ISO···β-CD complex in the five configurations (A–E).

Following the clustering analysis of MD snapshots, the selected ISO···β-CD configurations were subjected to geometry optimization and *E*_int_ calculations. Based on the calculated *E*_int_ values, the five configurations exhibiting the most negative *E*_int_ values were designated A–E and selected for further DFT analyses (Figure 2). The geometric parameters, interacting functional groups, and *E*_int_ values of these configurations are summarized in Table 1. The remaining configurations, along with their corresponding less negative *E*_int_ values, are presented in Figure S1, while side views of the optimized configurations A–E are provided in Figure S2. Furthermore, top and side views of the studied configurations A–E are presented using a tube representation in Figure S3.

As shown in Table 1 and Figure 2, configurations A–E exhibited relatively short intermolecular interaction distances and negative *E*_int_ values, indicating energetically favorable host–guest interactions within the ISO···β-CD complex. Illustratively, interaction distances ranged from 1.82 to 2.06 Å, suggesting the presence of short-range interactions that contributed to the stabilization of the ISO···β-CD complex. The data collected in Table 1 shows that configurations A, B, C, D, and E had *E*_int_ with values of −41.66, −38.77, −37.87, −37.13, and −33.88 kcal/mol, respectively. Given the obtained negative *E*_int_ values, the results highlighted the considerable potential of β-CD as an encapsulation platform for the ISO drug.

Consistent with the interacting groups identified in Table 1, the pyridine nitrogen of ISO interacted with the C6–OH groups at the narrower primary rim of β-CD, whereas the carbonyl and hydrazide groups interacted with the C2–OH and C3–OH groups at the wider secondary rim. Furthermore, the side views of configurations A–E (Figures S2 and S3) suggested that the pyridine ring was accommodated within the β-CD cavity, while the hydrazide moiety extended toward the wider cavity opening. These structural features supported the predicted inclusion orientation but did not establish the encapsulation pathway.

### 2.4. SAPT Calculation

To gain profound energy insights, SAPT analysis was substantially applied to the ISO∙∙∙β-CD complex in configurations A–E. The calculated values of *E*^SAPT0^ and its four respective components are presented in Table 2.

According to Table 2, the encapsulation process within the ISO∙∙∙β-CD complex in configurations A–E was energetically preferred, as evidenced by negative *E*^SAPT0^ values. Notably, the evaluated *E*^SAPT0^ values become more negative in a parallel way to *E*_int_ values (Table 1). As an illustration, configurations A, B, C, D, and E showed *E*^SAPT0^/*E*_int_ with values of −47.30/−41.66, −44.40/−38.77, −43.47/−37.87, −43.15/−37.13, and −38.42/−33.88 kcal/mol, respectively.

Among the SAPT energy components, *E*_elst_ exhibited the most negative values, identifying electrostatics as the dominant attractive contribution to ISO···β-CD stabilization. Notably, the magnitude of *E*_disp_ corresponded to approximately 71–81% of *E*_elst_, highlighting the substantial contributions of both electrostatic and dispersion interactions to the overall stabilization of the ISO···β-CD complex. Additionally, *E*_ind_ showed negative values, affirming its stabilizing role in the encapsulation process. However, positive values of *E*_exch_ signified its destabilizing role on the total energy (*E*^SAPT0^). As a numerical example, the *E*_elst_, *E*_ind_, *E*_disp_, and *E*_exch_ values for the ISO∙∙∙β-CD complex in configuration A were −47.47, −15.74, −33.58, and 49.48 kcal/mol, respectively. Overall, the negative *E*^SAPT0^ values indicated favorable short-range interactions in configurations A–E, arising from the combined contributions of *E*_elst_, *E*_ind_, and *E*_disp_, counterbalanced by *E*_exch_ repulsion.

### 2.5. QTAIM and NCI Calculations

**Figure 3:**
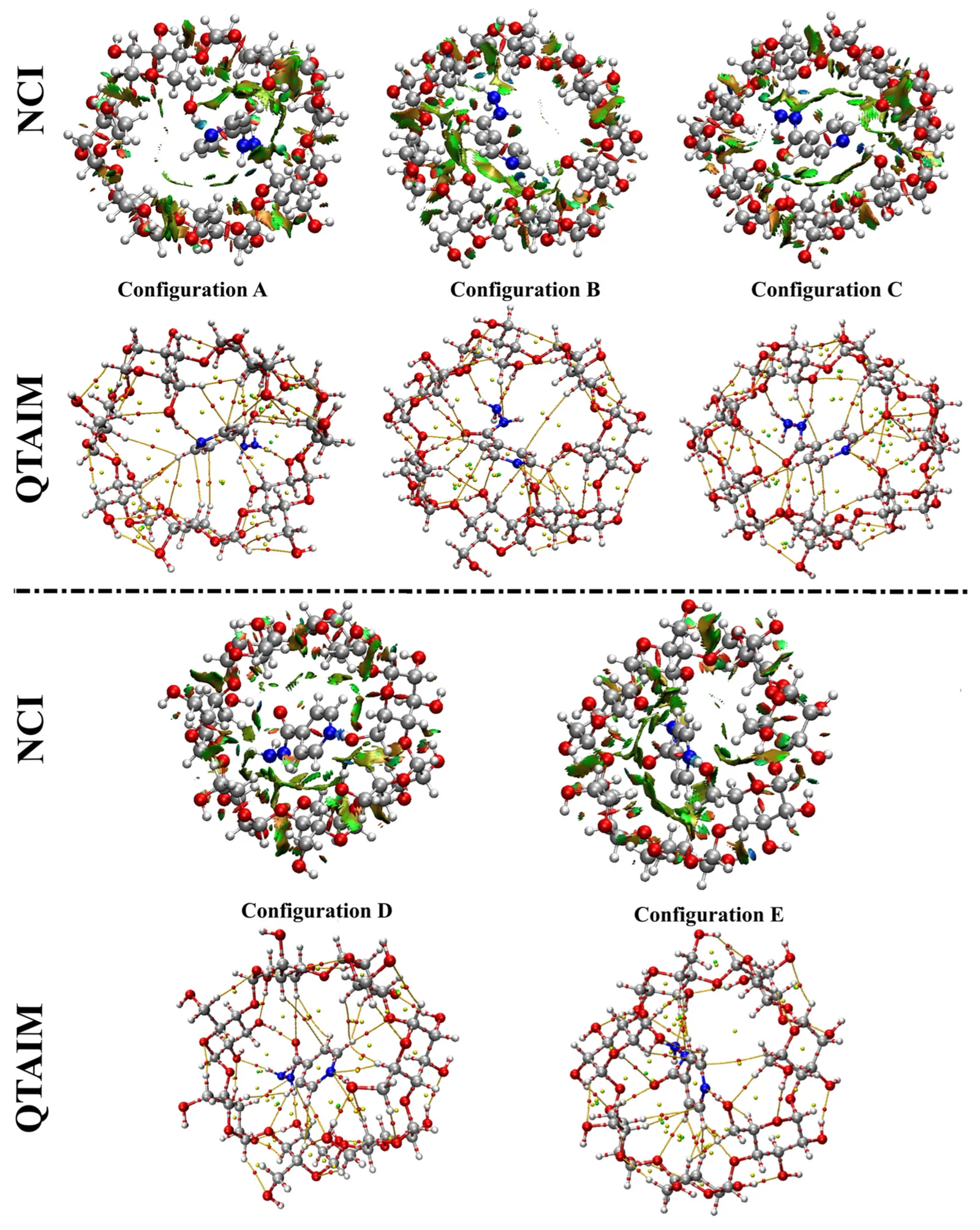
3D NCI and QTAIM plots for the ISO∙∙∙β-CD complex in configurations A–E. The NCI isosurfaces are colored based on (λ_2_)*ρ* values from −0.035 au (blue) to 0.020 au (red).

To gain deeper insights into intermolecular interactions within the ISO∙∙∙β-CD complex, QTAIM and NCI analyses were employed. The generated 3D QTAIM and NCI plots for the ISO∙∙∙β-CD complex in configurations A–E are presented in Figure 3. For further clarity, the 3D QTAIM and NCI plots are also presented using a licorice representation in Figure S4. In addition, the 2D NCI plots for the ISO∙∙∙β-CD complex in configurations A–E were generated and are presented in Figure S5. Furthermore, the calculated topological parameters at BCPs for the studied configurations are tabulated in Table 3.

As illustrated in Figure 3 and Figure S4, QTAIM analysis identified BPs and associated BCPs connecting ISO and β-CD, distinguishing intermolecular contacts from the intramolecular hydrogen-bonding network of β-CD. The identified contacts, including N···H–O, O···H–O, and N–H···O, supported the presence of hydrogen-bonding interactions contributing to the stabilization of the ISO···β-CD complex.

The 3D NCI isosurfaces (Figure 3 and Figure S4) provided complementary visualization of the noncovalent interaction regions. According to the NCI color scale, blue regions at more negative sign(λ_2_)*ρ* values indicated stronger attractive interactions, whereas green regions near zero reflected weaker, van der Waals-type contacts. Similarly, the two-dimensional NCI plots (Figure S5) exhibited low-RDG features at negative sign(λ_2_)*ρ* values across all configurations, further supporting the presence of attractive host–guest interactions.

As reported in Table 3, the positive values of H_b_ and ∇^2^*ρ*_b_ were consistent with predominantly closed-shell interactions within the ISO···β-CD complex. For instance, the O···H–O contacts in configurations A–E exhibited ∇^2^*ρ*_b_/H_b_ values of 0.1133/0.0028, 0.1247/0.0026, 0.1127/0.0024, 0.1193/0.0019, and 0.1003/0.0030 au, respectively. These findings indicated predominantly noncovalent interactions, consistent with the electrostatic contributions identified through SAPT analysis. Overall, the complementary QTAIM and NCI results supported the presence of attractive intermolecular interactions stabilizing the modeled ISO···β-CD complex.

### 2.6. Electronic Parameters

**Figure 4:**
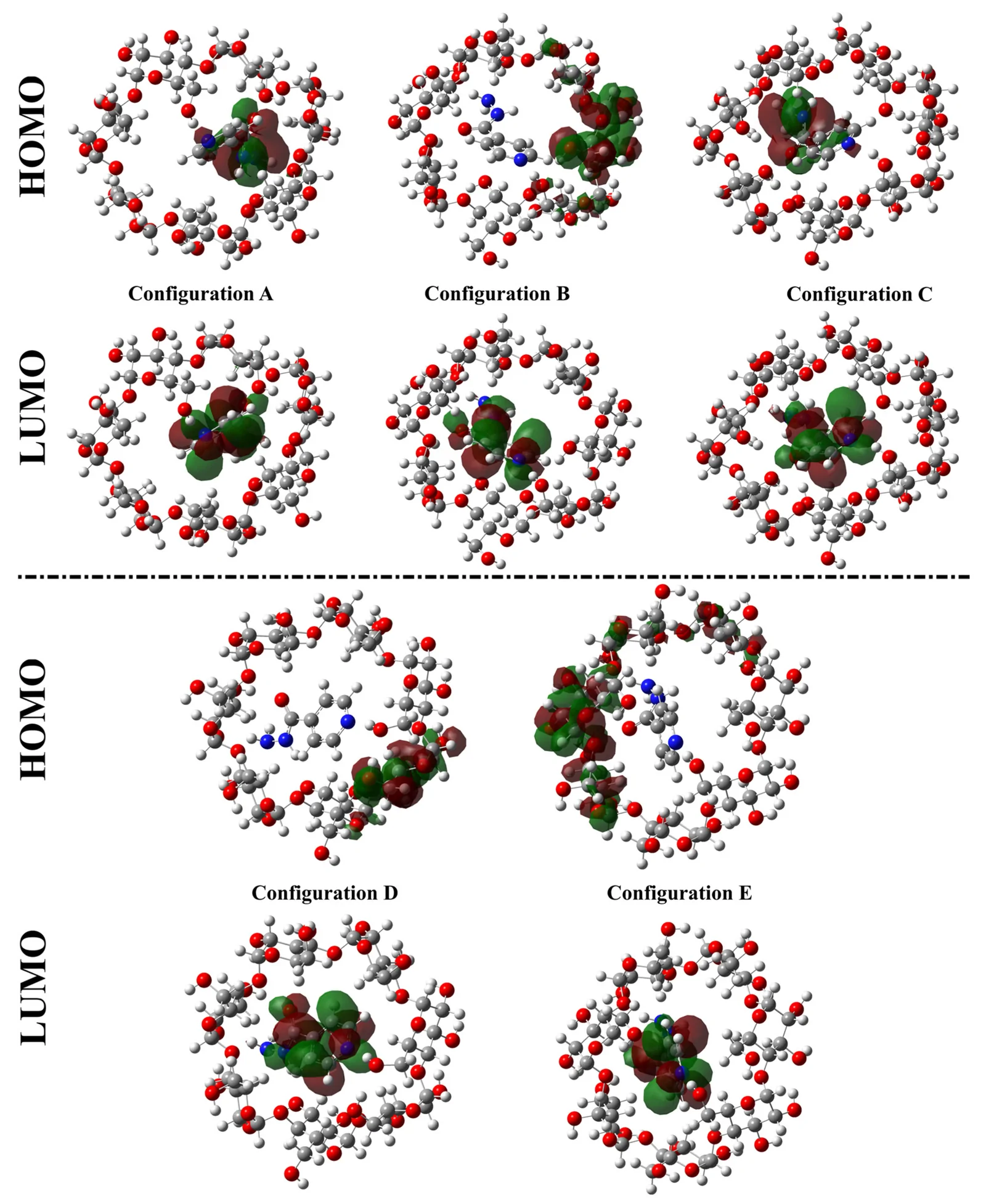
Visualization of HOMO and LUMO distributions for the ISO∙∙∙β-CD complex in configurations A–E.

For electronic investigation, the studied systems were subjected to FMO analysis. From FMO analysis, the HOMO/LUMO plots were extracted for the ISO∙∙∙β-CD complex in configurations A–E (Figure 4). For numerical illustration, the corresponding *E*_HOMO_, *E*_LUMO_, *E*_FL_, and *E*_gap_ values were calculated (Table 4).

Figure 4 illustrates the HOMO and LUMO distributions of the optimized ISO···β-CD complex in configurations A–E. The observed variations in frontier molecular orbital localization highlighted differences in the electronic structures of the investigated configurations, providing insights into their configuration-dependent electronic characteristics. The configuration-dependent HOMO localization arose from changes in the relative energies of host- and guest-centered orbitals; however, it did not independently establish orbital hybridization or charge transfer.

From the data in Table 4, the *E*_HOMO_ and *E*_LUMO_ values for ISO and β-CD notably changed following the encapsulation process within the ISO∙∙∙β-CD complex in configurations A–E. These variations indicated the ISO effect on the electronic features of β-CD. As an example, β-CD had *E*_HOMO_/*E*_LUMO_ values of −8.85/−0.74 eV, whereas after the encapsulation process, the ISO∙∙∙β-CD complex in configuration A showed −8.54/−1.19 eV. Consistent with *E*_HOMO_/*E*_LUMO_ changes, *E*_gap_ values for ISO and β-CD were also altered upon the encapsulation process. Illustratively, *E*_gap_ decreased from 8.11 eV for isolated β-CD to 7.36 eV for the ISO···β-CD complex in configuration A. Similar reductions in *E*_gap_ were observed across the remaining configurations, indicating narrower frontier orbital energy separations upon ISO encapsulation. In summary, the calculated parameters characterized the electronic properties of the ISO∙∙∙β-CD complex in configurations A–E.

### 2.7. Global Reactivity Indices

For further electronic characteristics, a series of global reactivity indices was computed for β-CD and ISO before and following the encapsulation process. The obtained *IP*, *EA*, *η*, and *S* parameters for β-CD, ISO, and the ISO∙∙∙β-CD complex in configurations A–E are collected in Table 5. Further parameters, including *µ* and *ω,* are gathered in Table S2.

In Table 5 and Table S2, electronic descriptors for β-CD notably varied following encapsulation within the ISO∙∙∙β-CD complex in configurations A–E, indicating a clear influence of ISO on the electronic properties of β-CD. For instance, β-CD had an *IP* value of 8.85 eV, which varied to 8.54 eV for the ISO∙∙∙β-CD complex in configuration A. Upon the encapsulation process, *S* values increased, suggesting greater electronic softness of the ISO∙∙∙β-CD complex. Numerically, the *S* value for β-CD was 0.25 eV^−1^, increasing to 0.27 eV^−1^ for the ISO∙∙∙β-CD complex in configuration A. Similarly, *η* values decreased following the encapsulation process, which consequently enhanced the electronic softness of the ISO∙∙∙β-CD complex in configurations A–E. For example, the *η* value for β-CD was 4.06 eV, decreasing to 3.68 eV for the ISO∙∙∙β-CD complex in configuration A. In summary, the calculated descriptors offered further insights into the electronic features of ISO, β-CD, and the ISO∙∙∙β-CD complex in configurations A–E.

### 2.8. TDOS Analysis

**Figure 5:**
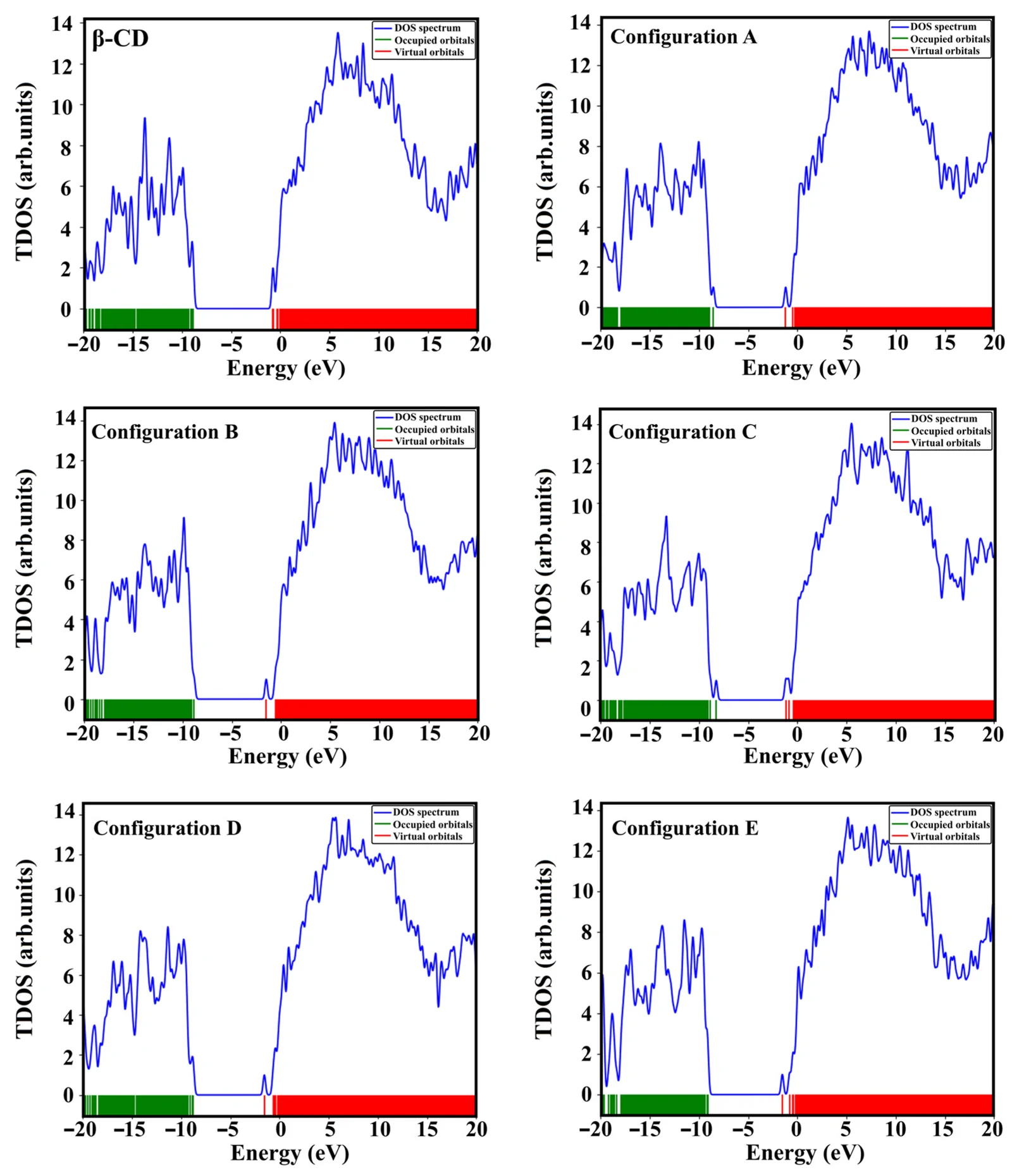
TDOS graphs for β-CD and the ISO∙∙∙β-CD complex in configurations A–E. The energy scale ranges from −20 to 20 eV.

Electronic features of the ISO∙∙∙β-CD complex were also examined in terms of TDOS analysis. Therefore, TDOS plots for β-CD and the ISO∙∙∙β-CD complex in configurations A–E were generated and are plotted in Figure 5.

The TDOS plots presented in Figure 5 revealed differences in the electronic structures of isolated β-CD and the ISO···β-CD complex, with additional peaks observed in configurations A–E, particularly within the −5 to 0 eV energy range. These features may be attributed to contributions from ISO molecular orbitals, although their definitive assignment requires further fragment-resolved DOS analysis. Furthermore, the *E*_gap_ of β-CD was noticeably changed following encapsulation within the ISO∙∙∙β-CD complex in configurations A–E (Figure 5). TDOS plots provided complementary electronic characterization but did not independently establish inclusion complexation or quantify interaction strength. Overall, the TDOS results highlighted changes in the electronic structure of β-CD upon ISO encapsulation, reflecting differences in the electronic characteristics of the resulting ISO···β-CD complex.

### 2.9. Solvent Effect

In relation to the key role of water in biological systems, the solvent effect on the stability of the encapsulation process within the ISO∙∙∙β-CD complex in configurations A–E was evaluated. Subsequently, their corresponding interaction ($E_{\mathrm{int}}^{\mathrm{solvent}}$) and solvation (Δ*E*_solv_) energy values were computed, as reported in Table 6.

As shown in Table 6, the negative $E_{\mathrm{int}}^{\mathrm{solvent}}$ values of the ISO···β-CD complex indicated energetically favorable electronic interactions across configurations A–E within the IEF-PCM implicit solvent framework. The calculated $E_{\mathrm{int}}^{\mathrm{solvent}}$ values ranged from −26.82 to −24.84 kcal/mol. Among the investigated configurations, configuration C exhibited the most negative $E_{\mathrm{int}}^{\mathrm{solvent}}$ value of −26.82 kcal/mol, indicating a change in energetic ranking relative to the gas-phase results. Notably, the consistently less negative $E_{\mathrm{int}}^{\mathrm{solvent}}$ values compared with their gas-phase counterparts suggested that implicit aqueous solvation weakened the calculated electronic interactions within the ISO···β-CD complex. Furthermore, configurations A–E exhibited negative Δ*E*_solv_ values ranging from −46.86 to −39.41 kcal/mol, indicating energetically favorable solvation of the preassembled complex. Overall, the IEF-PCM results supported favorable electronic interactions within the ISO···β-CD complex in an implicit aqueous environment, although these findings do not establish their thermodynamic binding favorability in solution.

### 2.10. Recovery Time

To preliminarily assess the potential release behavior of ISO from β-CD, the recovery-time (*τ*) values were estimated for configurations A–E at three temperatures (*T*_1_ = 298.15 K, *T*_2_ = 310.15, and *T*_3_ = 315.15 K). The calculated *τ* values are summarized in Table 7.

As shown in Table 7, the calculated *τ* values for the ISO∙∙∙β-CD complex in configurations A–E ranged from 3.12 × 10^18^ μs (at 298.15 K) to 2.89 × 10^11^ μs (at 315.15 K). The calculated *τ* values were directly correlated with the corresponding *E*_int_ values (Table 1), as the configuration exhibiting a more negative *E*_int_ value showed a longer estimated *τ* value. For numerical explanation, the ISO∙∙∙β-CD complex in configuration A had the most negative *E*_int_ (−41.66 kcal/mol) and correspondingly displayed the longest *τ* value (3.12 × 10^18^ μs at 298.15 K). At the examined temperatures, *τ* values decreased following the increase in *T*. For instance, configuration A had *τ* values of 3.12 × 10^18^, 2.06 × 10^17^, and 7.07 × 10^16^ μs at 298.15, 310.15, and 315.15 K, respectively.

In summary, these simplified DFT-based recovery-time calculations used the magnitude of the *E*_int_ as an approximation of the dissociation activation barrier and assumed an attempt frequency of 10^18^ s^−1^ without system-specific validation. Furthermore, the aqueous dissociation pathway, solvent reorganization, and entropic contributions were not explicitly considered. These calculated *τ* values should be interpreted cautiously because they do not represent validated predictions of the actual ISO release times from β-CD. Accordingly, no conclusions regarding drug-release kinetics or pharmaceutical performance can be drawn from these estimates.

### 2.11. Adsorption Process

The adsorption of ISO onto the external surface of β-CD was further investigated using the same computational approach employed for the encapsulation model, involving MD simulation, cluster analysis, and subsequent DFT calculations. Unlike the encapsulation model, in which ISO was initially positioned within the β-CD cavity, the adsorption model considered ISO initially placed on the external surface of β-CD.

**Figure 6:**
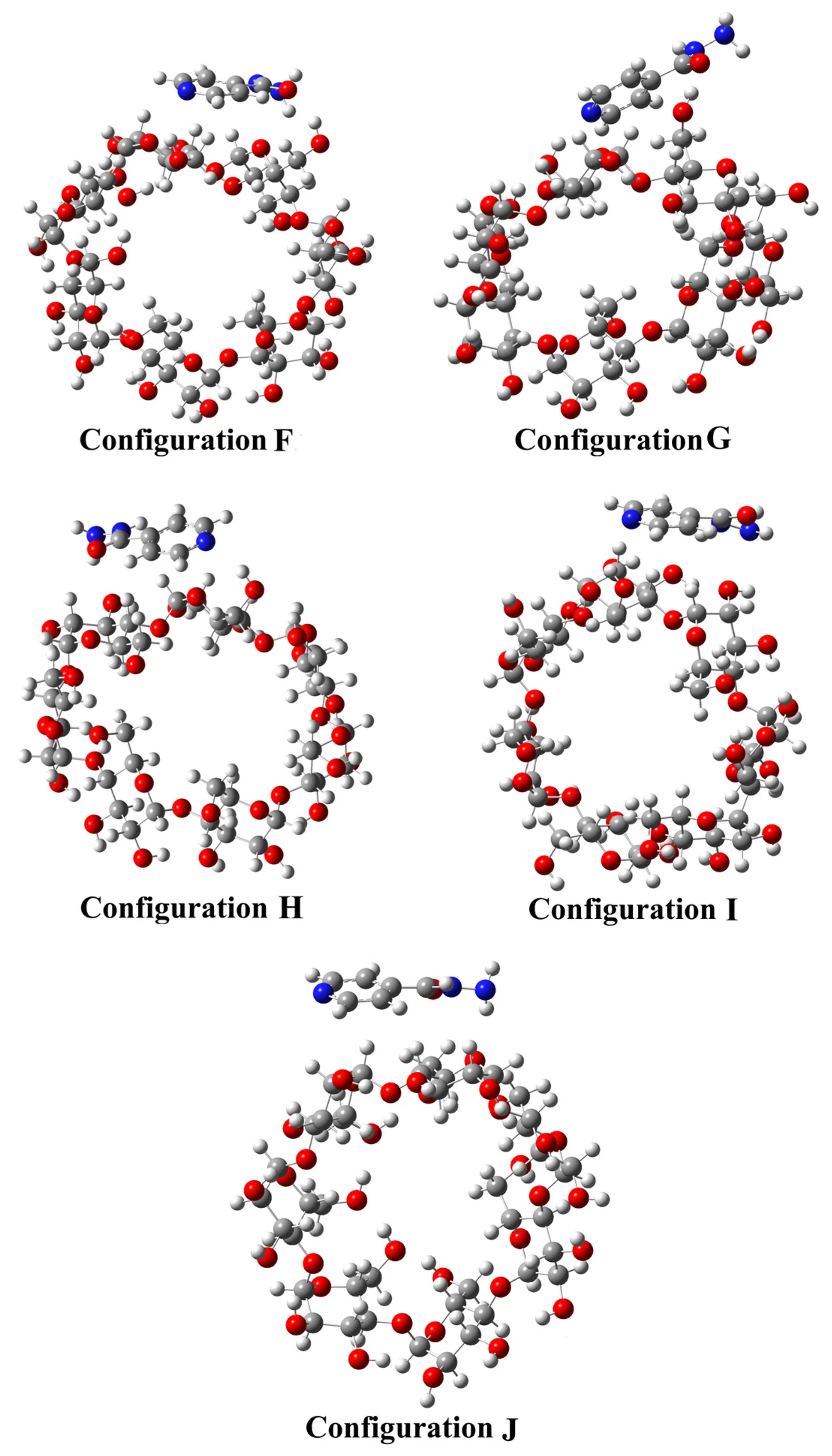
Optimized structures for the ISO∙∙∙β-CD complex in configurations F–J.

The optimized ISO∙∙∙β-CD complexes in configurations F–J are gathered in Figure 6, and their related *E*_int_, $E_{\mathrm{int}}^{\mathrm{solvent}}$, and Δ*E*_solv_ values were calculated (Table 8).

As illustrated in Figure 6, the MD-derived adsorption configurations (F–J) revealed that ISO remained outside the β-CD cavity, in proximity to its hydroxyl-bearing regions. These configurations suggested potential intermolecular interactions between the polar functional groups of ISO and the accessible hydroxyl groups of β-CD.

As illustrated in Table 8, the ISO drug was favorably adsorbed over the β-CD surface in configurations F–J, with negative *E*_int_ values between −18.42 and −10.70 kcal/mol. Comparatively, the encapsulation configurations (A–E) exhibited more favorable electronic interactions than the adsorption configurations (F–J), as indicated by their more negative *E*_int_ values. For example, configuration A had an *E*_int_ value of −41.66 kcal/mol in the case of the encapsulation process, whereas the value became less negative, reaching −18.42 kcal/mol for configuration F, in the adsorption model. Notably, similar behavior was observed in the water effect calculations for the adsorption process relative to the encapsulation process. Illustratively, $E_{\mathrm{int}}^{\mathrm{solvent}}$ values for configurations A and F were −25.92 and −13.21 kcal/mol in the case of the encapsulation and adsorption processes, respectively. These observations indicated that the encapsulation of ISO into the cavity of β-CD was highly preferable over its adsorption model.

### 2.12. Study Limitation and Future Perspectives

The present MD-assisted DFT study provided molecular-level insights into the structural, energetic, and electronic characteristics of ISO···β-CD complexes. Nevertheless, several limitations should be acknowledged, including the limited MD simulation timescale, absence of independent simulation replicates, force-field dependence, assumed 1:1 host–guest stoichiometry, and use of the IEF-PCM implicit solvation model. In particular, the 10 ns MD simulations were primarily intended to explore local host–guest conformations and identify representative configurations for subsequent DFT calculations, rather than to establish conformational convergence, spontaneous encapsulation, or equilibrium binding. Moreover, the adopted stoichiometry represented a modeling assumption rather than an experimentally established composition. The calculated electronic interaction energies do not directly represent binding free energies and cannot independently establish binding affinity or release kinetics. Although SAPT analysis identified the major energetic contributions, further fragment-resolved analysis would be required to quantify individual functional-group contributions. Similarly, the simplified recovery-time estimates were based on assumed dissociation barriers and attempt frequencies, without explicitly accounting for solvent reorganization or entropic contributions. Accordingly, the findings should be interpreted as preliminary theoretical evidence that requires further computational and experimental validation. Future investigations involving extended MD simulations, independent replicates, and more rigorous binding free-energy calculations would provide a more comprehensive assessment of host–guest stability and encapsulation behavior. Furthermore, experimental evaluation of complex formation, drug-loading efficiency, release characteristics, toxicity, biocompatibility, and pharmacokinetic properties is essential to establish the pharmaceutical potential of the ISO···β-CD system.

## 3. Materials and Methods

The encapsulation potential of β-CD toward the ISO drug was systematically elucidated by performing MD simulation followed by DFT computations. Using Gaussian 09 software [45], DFT computations were conducted with the M06-2X method and 6-311+G** basis set [46,47]. The utilized method and basis set were selected based on the literature evidence for their thorough characterization of important parameters relevant to the drug delivery process [48,49,50]. The UltraFine numerical integration grid was employed throughout the calculations. The initial structure of β-CD was obtained from the literature [27], followed by geometry optimization of both β-CD and ISO. Given the optimized β-CD and ISO, the ESP analysis was implemented; then, molecular electrostatic potential (MEP) maps were generated on a 0.002 au electron density isosurface. To quantify the MEP findings, the values of electrostatic potential extrema (i.e., *V*_s,min_ and *V*_s,max_) were determined by utilizing Multiwfn 3.7 software [51].

MD simulation was performed using the AMBER20 package [52] to investigate the conformational behavior and host–guest interactions of the 1:1 ISO···β-CD complex. Two distinct initial arrangements were considered, with ISO positioned either inside the β-CD cavity (encapsulation) or outside the cavity (adsorption). The initial 1:1 ISO···β-CD complex was constructed by positioning ISO within the β-CD cavity, with its pyridine moiety directed toward the primary rim (C6–OH) and its hydrazide group oriented toward the secondary rim (C2/C3–OH). An additional configuration was prepared by initially placing ISO outside the β-CD cavity to investigate external adsorption. Both ISO and β-CD were modeled in their neutral states (net charge = 0 for each molecule), with their force-field parameters assigned using the General AMBER Force Field 2 (GAFF2) [53,54]. The partial atomic charges of ISO and β-CD were evaluated via the restrained electrostatic potential (RESP) approach, employing HF/6-31G* calculations for electrostatic potential fitting [55]. Within an octahedral TIP3P water box, the ISO⋯β-CD complex was dissolved using a distance of 1.2 nm between the solute and the box boundaries. The solvated ISO⋯β-CD complex was energetically minimized for 5000 cycles using steepest descent and conjugate gradient methods. Thereafter, the minimized ISO⋯β-CD complex was heated up to 310 K under NVT conditions and subsequently equilibrated for 1 ns under NPT conditions. For the obtained complex, the production step ran over 10 ns, and snapshots were extracted per 10 ps, yielding a total of 1000 snapshots. Production MD simulation was conducted using a 2 fs integration timestep, with the temperature maintained at 310 K using a Langevin thermostat with a collision frequency of 2 ps^−1^. Pressure was maintained at 1 atm using a Berendsen barostat with isotropic position scaling [56]. A nonbonded cutoff of 10 Å was employed, while long-range electrostatic interactions were treated using the particle-mesh Ewald (PME) method [57]. Following the removal of water molecules and counterions, conformational clustering of the MD trajectory was performed using the hierarchical agglomerative clustering algorithm with average linkage, as implemented in CPPTRAJ [52]. Structural similarity was assessed using the root-mean-square deviation (RMSD), with a clustering threshold of 2.0 Å. Within each cluster, conformation with the lowest molecular mechanics potential energy was selected as the representative configuration. The selected configurations were subsequently subjected to DFT geometry optimization and interaction energy calculations to evaluate the energetic favorability of different ISO···β-CD configurations. The MD simulations were primarily designed to explore the local conformational arrangements of the preassembled encapsulation and adsorption systems and provide representative structures for further quantum chemical investigation, rather than to establish conformational convergence, spontaneous encapsulation, or equilibrium binding.

The resulting ISO⋯β-CD complex in various configurations was initially optimized using M06-2X/6-311+G** level of theory. Given the optimized structures, interaction (*E*_int_) energy values were calculated as shown below:

*E*_int_ = *E*_ISO∙∙∙β−CD_ − (*E*_β−CD in complex_ + *E*_ISO in complex_) + *E*_BSSE_ (1)

where *E*_ISO∙∙∙β−CD_ identifies the energy of the ISO⋯β-CD complex and *E*_β−CD in complex_ and *E*_ISO in complex_ are the energies of β-CD and ISO, respectively. Both β-CD and ISO energies were evaluated at their geometries extracted from the optimized complex. The reported *E*_int_ values excluded fragment deformation energies, thermal and entropic corrections, conformational averaging, and solvation/desolvation contributions. Accordingly, *E*_int_ represented electronic interaction energies and should not be interpreted as solution-phase binding free energies or quantitative measures of binding affinity. Basically, the counterpoise correction procedure was considered in energy calculations, which in turn eliminated the basis set superposition error (BSSE) [58].

To evaluate individual force contributions to stabilize the overall encapsulation process within the ISO⋯β-CD complex, SAPT analysis was conducted through the PSI4 package [59]. SAPT0 calculation employed the 6-311+G** basis set with a restricted Hartree–Fock reference and density fitting for the SCF procedure. The SCF energy and density convergence thresholds were set to 10^−5^. For the studied configurations, the total energy (*E*^SAPT0^) values were first calculated based on the SAPT0 level according to the following formula [60]:

*E*^SAPT0^ = *E*_elst_ + *E*_exch_ + *E*_ind_ + *E*_disp_ (2)

As outlined above, the *E*^SAPT0^ contained four forces: electrostatic (*E*_elst_), induction (*E*_ind_), dispersion (*E*_disp_), and exchange (*E*_exch_). Each force could be individually computed as described below:

(3) $$E_{\mathrm{exch}}=\;E_{\mathrm{exch}}^{\left(10\right)}$$

(4) $$E_{\mathrm{elst}}=E_{\mathrm{elst}}^{\left(10\right)}$$

(5) $$E_{\mathrm{ind}}=E_{\mathrm{ind},\mathrm{resp}}^{\left(20\right)}+E_{\mathrm{exch}-\mathrm{ind},\mathrm{resp}}^{\left(20\right)}+{\delta E}_{\mathrm{HF},\mathrm{resp}}^{\left(2\right)}$$

(6) $$E_{\mathrm{disp}}=E_{\mathrm{disp}}^{\left(20\right)}+E_{\mathrm{exch}-\mathrm{disp}}^{\left(20\right)}$$

In Equations (3)–(6), the superscripts (10) and (20) denote first- and second-order contributions with respect to the intermolecular interaction potential, respectively, with both evaluated at zeroth order in intramonomer electron correlation. The subscript *resp* indicates the inclusion of orbital relaxation effects, whereas ${\delta E}_{\mathrm{HF}}^{\left(2\right)}$ accounts for higher-order induction contributions estimated from Hartree–Fock calculations.

QTAIM and NCI analyses were conducted to systematically characterize the interactions within the ISO⋯β-CD complex using Multiwfn 3.7 software [51]. QTAIM analysis revealed the interacted species through generated bond paths/bond critical points (BPs/BCPs), whereas NCI analysis elaborated the interactions through the related colored isosurfaces. The NCI isosurfaces were colored based on (λ_2_)*ρ* values from −0.035 au (blue) to 0.020 au (red). To visualize QTAIM and NCI plots, the Visual Molecular Dynamics (VMD) program was utilized [61]. As numerical evidence, QTAIM-topological parameters at the considered BCPs were calculated, including electron density (*ρ*_b_), total energy density (H_b_), and Laplacian (∇^2^*ρ*_b_).

To provide a comprehensive electronic elucidation, FMO analysis was carried out, in which the highest occupied and lowest unoccupied molecular orbitals (i.e., HOMO and LUMO, respectively) were mapped. With respect to FMO analysis, corresponding *E*_HOMO_ and *E*_LUMO_ values were also computed. Thereafter, energy gap (*E*_gap_) values were evaluated for the studied systems, using the following formula:

*E*_gap_ = *E*_LUMO_ − *E*_HOMO_ (7)

In addition, the ionization potential (*IP*) and electron affinity (*EA*) were approximated from *E*_HOMO_ and *E*_LUMO_, respectively, using a Koopmans-type approach, as follows:

*IP* ≈ −*E*_HOMO_ (8)

*EA* ≈ −*E*_LUMO_ (9)

Fermi level (*E*_FL_) values were also computed using *E*_HOMO_/*E*_LUMO_ values, as outlined below:

(10) $$E_{\mathrm{FL}}=E_{\mathrm{HOMO}}+\frac{E_{\mathrm{LUMO}}-E_{\mathrm{HOMO}}}{2}$$

Furthermore, global descriptors, including global hardness (*η*), global softness (*S*), chemical potential (*μ*), and electrophilicity index (*ω*), were calculated by the following equations:

(11) $$\eta =\frac{E_{\mathrm{LUMO}}-E_{\mathrm{HOMO}}}{2}$$

(12) $$S=\frac{1}{\eta }$$

(13) μ=ELUMO+EHOMO 2

(14) $$\omega =\frac{\mu^{2}}{2\eta }$$

Considering the electronic aspects, TDOS analysis was additionally performed using GaussSum software [62]. To assess the water effect on the stability of the interactions within the ISO⋯β-CD complex, the integral equation formalism polarizable continuum model (IEF-PCM) was utilized [63,64,65]. Following M06-2X/6-311+G** level of theory, the ISO⋯β-CD complex in configurations A–E was subjected to geometrical optimization considering the IEF-PCM framework. Interaction energies in the water solvent ($E_{\mathrm{int}}^{\mathrm{solvent}}$) were subsequently calculated for the optimized configurations using the same equation utilized in the gas phase (Equation (1)). Moreover, solvation energies (Δ*E*_solv_) were estimated by considering the total energy of the complex in water and its related energy in the gas phase, as outlined below:

Δ*E*_solv_ = *E*_solvent_ − *E*_gas_ (15)

Finally, ISO dissociation from β-CD was preliminarily evaluated using simplified recovery-time (*τ*) estimates, assuming an attempt frequency (*v* = 10^18^ s^−1^) [66,67] and the universal gas constant (R = 0.001987 kcal/mol.K), as expressed below:

(16) $$\tau =v^{-1}\exp\left(\frac{-E_{\mathrm{int}}}{\mathrm{RT}}\right)$$

As expressed in Equation (16), the gas-phase *E*_int_ was used as an assumed energy parameter for estimating the *τ* values; however, it was not established as the activation energy barrier for ISO dissociation from β-CD in aqueous solution. The calculation also does not explicitly account for solvent reorganization, entropic contributions, or other factors that may influence the dissociation process. Consequently, the calculated *τ* values should be regarded as model-dependent illustrative estimates rather than physically validated predictions of ISO release times. They should not be used to infer actual release kinetics or pharmaceutical performance. Reliable predictions would require a more rigorous treatment of the dissociation free-energy barrier in an appropriate solvent environment together with experimental validation.

The Cartesian coordinates of the optimized ISO and β-CD, along with their related ISO⋯β-CD complex in configurations A–J, are provided in Table S1.

## 4. Conclusions

The encapsulation potential of β-CD toward the ISO drug was theoretically examined by MD simulation and DFT calculations. From clustering analysis of MD snapshots, the encapsulation process within the ISO⋯β-CD complex in various configurations was extensively examined. Negative interaction energy (*E*_int_) values were obtained for the ISO∙∙∙β-CD complex, highlighting favorable electronic interactions in the examined configurations. Configuration A showed a significant negative *E*_int_ with a value of –41.66 kcal/mol. SAPT analysis illustrated the stabilizing contribution of electrostatic, induction, and dispersion forces in the overall encapsulation process within the ISO∙∙∙β-CD complex. QTAIM and NCI analyses demonstrated the attractive interactions within the ISO∙∙∙β-CD complex in all examined configurations. These findings indicated that electrostatic attraction, hydrogen-bonding contacts, and substantial dispersion contributions stabilized the ISO⋯β-CD complex. Furthermore, electronic characterization of the encapsulation process within the ISO∙∙∙β-CD complex was elucidated by means of FMO and TDOS analyses. The water effect on the interactions within the ISO⋯β-CD complex was investigated. Moreover, simplified recovery-time estimates were calculated for ISO from β-CD. Comparatively, the adsorption of ISO over β-CD was examined and observed to be less favorable than the encapsulation model. Overall, the results supported the structural stability and energetic feasibility of ISO encapsulation within β-CD; however, experimental validation is required to establish the pharmaceutical performance of the ISO∙∙∙β-CD complex under biological conditions.

## Figures and Tables

**Table 1:** Calculated bond parameters, interacting groups, and interaction (*E*_int_) energy values for the ISO∙∙∙β-CD complex in configurations A–E.

Configuration	Bond Parameters		Interacting Groups		*E*_int_ (kcal/mol)
	Distance (Å)	Angle (°)	ISO	β-CD	
A	O∙∙∙H (1.87)	O∙∙∙H–O (154.47)	Carbonyl O	C3–OH/secondary	−41.66
	H∙∙∙O (1.99)	N–H∙∙∙O (137.17)	Hydrazide –CO–NH–	C3–OH/secondary	
	N∙∙∙H (2.06)	N∙∙∙H–O (162.10)	Pyridine N	C6–OH/primary	
B	O∙∙∙H (1.82)	O∙∙∙H–O (158.60)	Carbonyl O	C3–OH/secondary	−38.77
	N∙∙∙H (1.99)	N∙∙∙H–O (169.86)	Pyridine N	C6–OH/primary	
	H∙∙∙O (2.00)	N–H∙∙∙O (143.77)	Terminal –NH_2_	C2–OH/secondary	
C	O∙∙∙H (1.86)	O∙∙∙H–O (164.50)	Carbonyl O	C3–OH/secondary	−37.87
	H∙∙∙O (1.92)	N–H∙∙∙O (151.91)	Hydrazide –CO–NH–	C3–OH/secondary	
	N∙∙∙H (1.96)	N∙∙∙H–O (152.48)	Pyridine N	C6–OH/primary	
D	O∙∙∙H (1.84)	O∙∙∙H–O (155.66)	Carbonyl O	C3–OH/secondary	−37.13
	N∙∙∙H (1.92)	N∙∙∙H–O (170.67)	Pyridine N	C6–OH/primary	
	H∙∙∙O (1.93)	N–H∙∙∙O (160.67)	Hydrazide –CO–NH–	C3–OH/secondary	
E	O∙∙∙H (1.93)	O∙∙∙H–O (152.45)	Carbonyl O	C3–OH/secondary	−33.88
	H∙∙∙O (2.05)	N–H∙∙∙O (142.05)	Terminal –NH_2_	C2–OH/secondary	
	N∙∙∙H (2.06)	N∙∙∙H–O (161.00)	Pyridine N	C6–OH/primary	

**Table 2:** Total energy (*E*^SAPT0^), electrostatic (*E*_elst_), induction (*E*_ind_), dispersion (*E*_disp_), and exchange (*E*_exch_) energy values for the ISO∙∙∙β-CD complex in configurations A–E. Obtained energies are given in kcal/mol.

Configuration	*E* ^SAPT0^	*E*_elst_	*E*_ind_	*E*_disp_	*E*_exch_
A	−47.30	−47.47	−15.74	−33.58	49.48
B	−44.40	−50.95	−16.40	−37.48	60.43
C	−43.47	−44.55	−15.57	−33.57	50.22
D	−43.15	−49.12	−17.15	−34.34	57.47
E	−38.42	−45.06	−13.05	−36.31	55.99

**Table 3:** Computed topological parameters at BCPs for the ISO∙∙∙β-CD complex in configurations A–E.

Configuration	Interaction	*ρ*_b_ (au)	∇^2^*ρ*_b_ (au)	H_b_ (au)
A	O∙∙∙H-O	0.0257	0.1133	0.0028
	N-H∙∙∙O	0.0231	0.0951	0.0027
	N∙∙∙H-O	0.0221	0.0752	0.0019
B	O∙∙∙H-O	0.0285	0.1247	0.0026
	N∙∙∙H-O	0.0269	0.0863	0.0011
	N-H∙∙∙O	0.0228	0.0902	0.0024
C	O∙∙∙H-O	0.0269	0.1127	0.0024
	N-H∙∙∙O	0.0248	0.1048	0.0029
	N∙∙∙H-O	0.0289	0.0914	0.0004
D	O∙∙∙H-O	0.0293	0.1193	0.0019
	N∙∙∙H-O	0.0311	0.0960	−0.0001
	N-H∙∙∙O	0.0174	0.0633	0.0019
E	O∙∙∙H-O	0.0228	0.1003	0.0030
	N-H∙∙∙O	0.0207	0.0804	0.0023
	N∙∙∙H-O	0.0234	0.0771	0.0016

**Table 4:** Calculated electronic parameters for β-CD and ISO, along with the ISO∙∙∙β-CD complex in configurations A–E.

System	Configuration	*E*_HOMO_ (eV)	*E*_LUMO_ (eV)	*E*_gap_ (eV)	*E*_FL_ (eV)
**β-CD**		−8.85	−0.74	8.11	−4.80
**ISO**		−9.11	−1.00	8.12	−5.06
**ISO∙∙∙β-CD**	A	−8.54	−1.19	7.36	−4.86
	B	−8.84	−1.50	7.35	−5.17
	C	−8.25	−1.17	7.08	−4.71
	D	−8.77	−1.54	7.23	−5.15
	E	−9.11	−1.52	7.59	−5.32

**Table 5:** Calculated *IP*, *EA*, *η*, and *S* parameters for β-CD, ISO, and the ISO∙∙∙β-CD complex in configurations A–E.

System	Configuration	*IP* (eV)	*EA* (eV)	*η* (eV)	*S* (eV^−1^)
**β-CD**		8.85	0.74	4.06	0.25
**ISO**		9.11	1.00	4.06	0.25
**ISO∙∙∙β-CD**	A	8.54	1.19	3.68	0.27
	B	8.84	1.50	3.67	0.27
	C	8.25	1.17	3.54	0.28
	D	8.77	1.54	3.61	0.28
	E	9.11	1.52	3.79	0.26

**Table 6:** Interaction ($E_{\mathrm{int}}^{\mathrm{solvent}}$) and solvation (Δ*E*_solv_) energy values for the ISO∙∙∙β-CD complex in configurations A–E.

Configuration	$E_{\mathrm{int}}^{\mathrm{solvent}}$ (kcal/mol)	Δ*E*_solv_ (kcal/mol)
A	−25.92	−39.41
B	−25.20	−46.48
C	−26.82	−44.68
D	−25.78	−40.43
E	−24.84	−46.86

**Table 7:** Computed recovery-time (*τ*, μs) values for the ISO∙∙∙β-CD complex in configurations A–E.

Configuration	*τ*		
	*T*_1_ = 298.15 K	*T*_2_ = 310.15 K	*T*_3_ = 315.15 K
A	3.12 × 10^18^	2.06 × 10^17^	7.07 × 10^16^
B	2.39 × 10^16^	1.91 × 10^15^	7.04 × 10^14^
C	5.25 × 10^15^	4.44 × 10^14^	1.68 × 10^14^
D	1.51 × 10^15^	1.34 × 10^14^	5.15 × 10^13^
E	6.30 × 10^12^	6.92 × 10^11^	2.89 × 10^11^

**Table 8:** Interaction (*E*_int_) energies in the gas phase and interaction ($E_{\mathrm{int}}^{\mathrm{solvent}}$) and solvation (Δ*E*_solv_) energies in the water phase for the adsorption process within the ISO∙∙∙β-CD complex in configurations F–J.

Configuration	*E*_int_ (kcal/mol)	$E_{\mathrm{int}}^{\mathrm{solvent}}$ (kcal/mol)	Δ*E*_solv_ (kcal/mol)
F	−18.42	−13.21	−49.84
G	−18.16	−14.16	−49.21
H	−16.30	−10.32	−43.82
I	−14.02	−9.24	−47.71
J	−10.70	−6.68	−46.90

## Data Availability

The data that supports the findings of this study are available in the Supplementary Material of this article.

## Supplementary Materials

The following supporting information can be downloaded at: https://www.mdpi.com/article/10.3390/ijms27219838/s1.
