# Supplementary material for: Molecular Modeling of Isoniazid Encapsulation by β-Cyclodextrin for Drug Delivery: Molecular Dynamics, Interaction Energies, and DFT Insights
Source: Int J Mol Sci. 2026 Nov 3;27(21):9838. doi: 10.3390/ijms27219838 (PMC13763234; doi:10.3390/ijms27219838)
Supplement: Supplementary file 1 [file ijms-27-09838-s001.zip › ijms-4621366-supplementary.pdf]

<u>Table of Contents:</u>

Content	Page Number
**Figure S1.** Optimized Isoniazid (ISO)···β-cyclodextrin (β-CD) complex in various configurations. Interaction energies (E_{int}) are in kcal/mol.	**S2**
**Figure S2.** Side views of optimized structures for ISO···β-CD complex in configurations A–E.	**S3**
**Figure S3.** (a) Top and (b) side views of the optimized ISO···β-CD complex structures in configurations A–E, displayed in tube representation.	**S4**
**Figure S4.** 3D NCI and QTAIM plots, shown in licorice representation, for ISO···β-CD complex in configurations A–E.	**S5**
**Figure S5.** 2D NCI plots of ISO···β-CD complex in configurations A–E.	**S6**
**Table S1.** Cartesian atomic coordinates for isolated ISO and β-CD, along with their related ISO···β-CD complex in configurations A–J.	**S7**
**Table S2.** Calculated μ and ω parameters for β-CD, ISO, and ISO···β-CD complex in configurations A–E.	**S51**

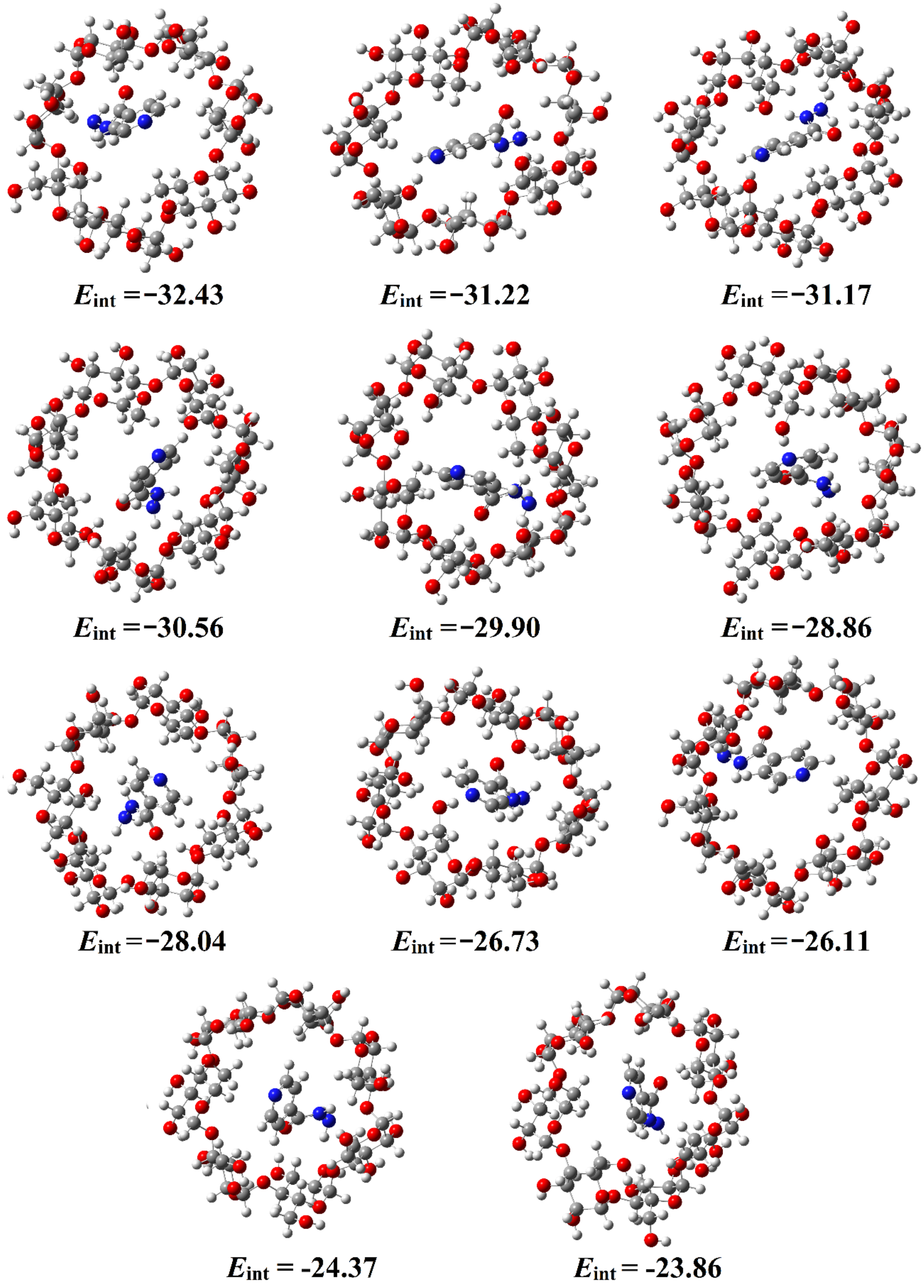

Figure S1. Optimized Isoniazid (ISO)···β-cyclodextrin (β-CD) complex in various configurations. Interaction energies (E_{int}) are in kcal/mol.

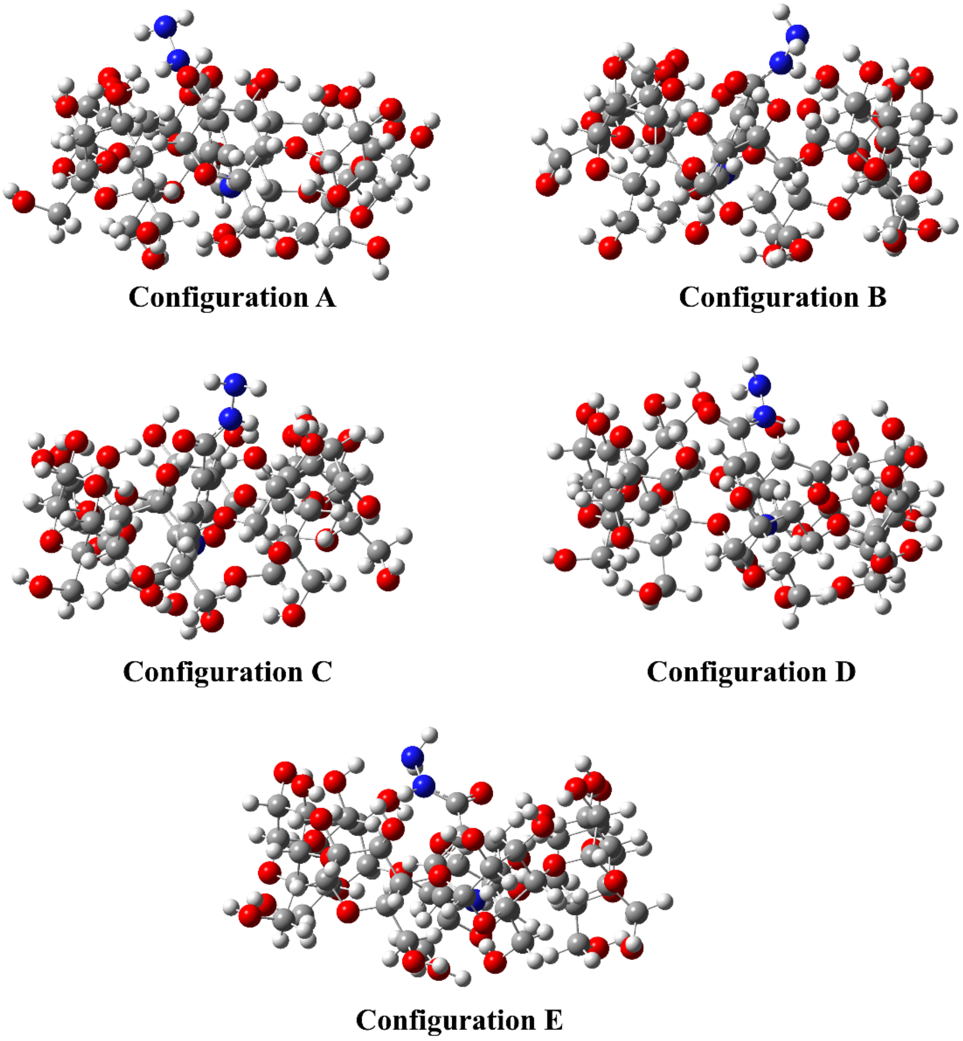

Figure S2. Side views of optimized structures for ISO···β-CD complex in configurations A–E.

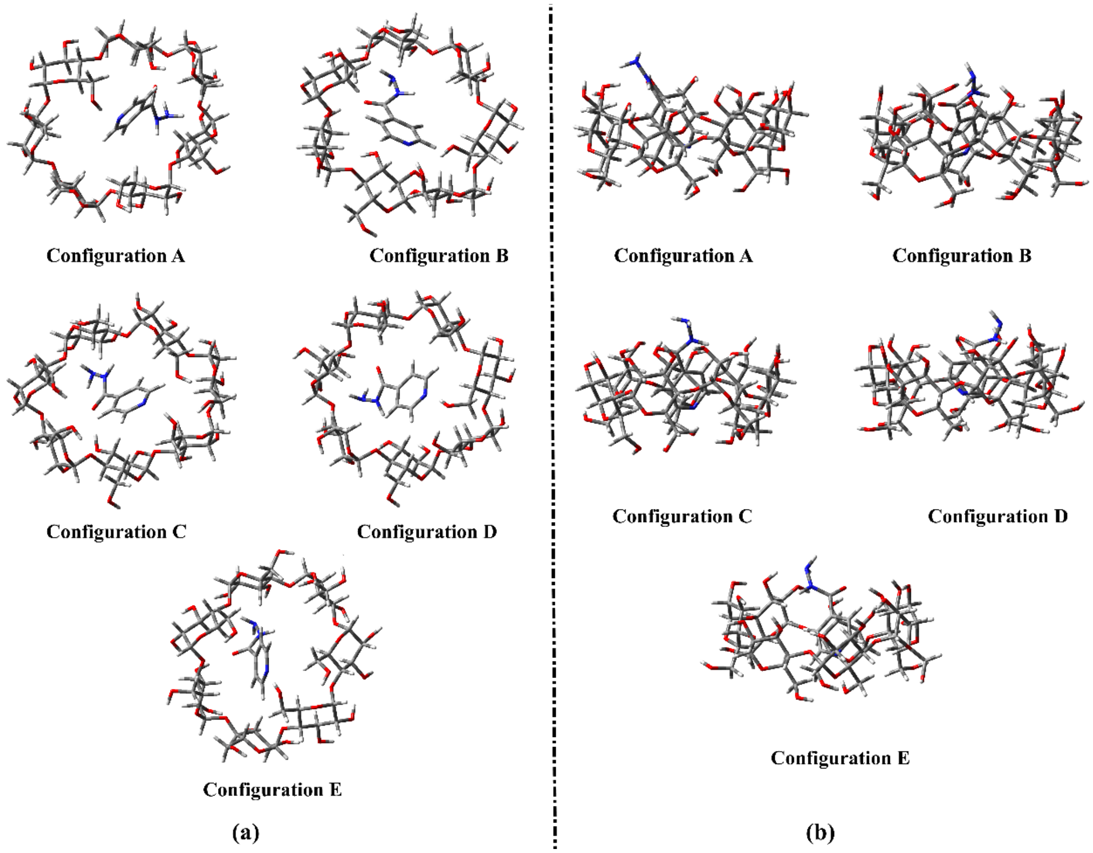

Figure S3. (a) Top and (b) side views of the optimized ISO···β-CD complex structures in configurations A–E, displayed in tube representation.

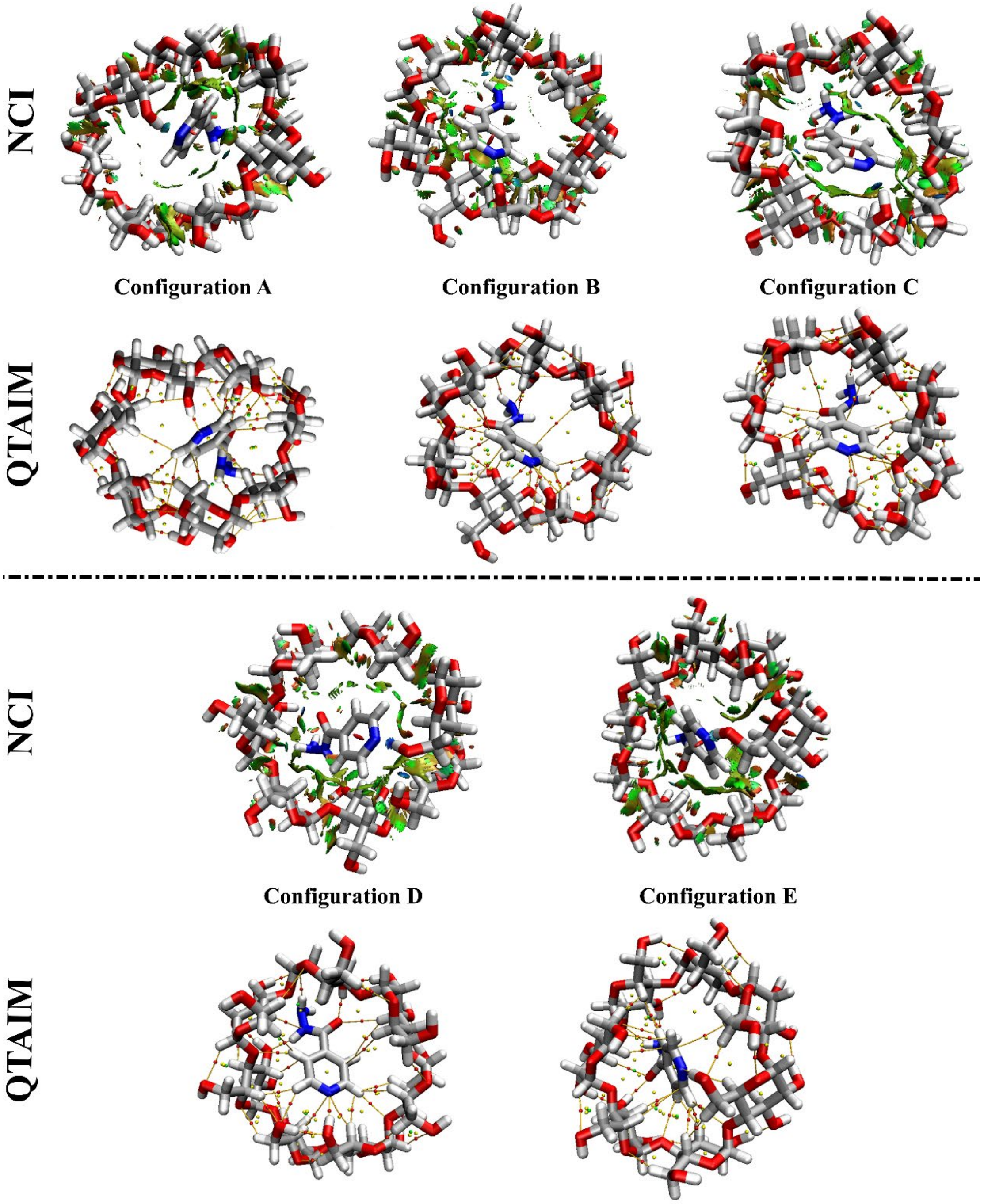

Figure S4. 3D NCI and QTAIM plots, shown in licorice representation, for ISO···β-CD complex in configurations A–E.

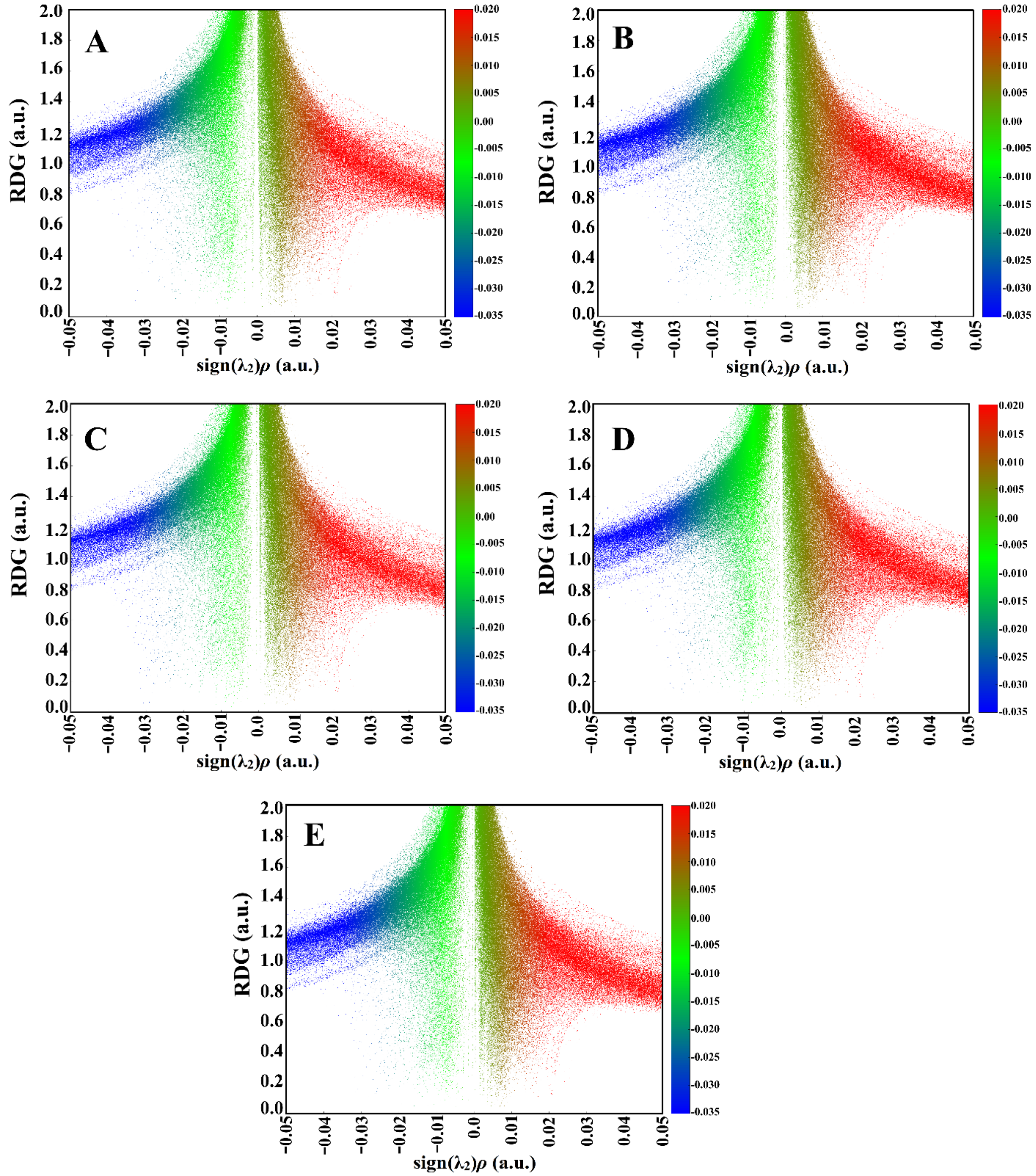

Figure S5. 2D NCI plots of ISO···β-CD complex in configurations A–E.

Table S1. Cartesian atomic coordinates for isolated ISO and β-CD, along with their related ISO···β-CD complex in configurations A–J.

ISO

Cartesian atomic coordinates:

C	0.77997400	-0.82997900	0.79229700
C	1.35981400	-0.48187100	3.08521300
C	-0.17941000	-1.73471800	1.23094600
C	0.37746600	-1.41147000	3.41421300
C	1.55921400	-0.17884000	1.74158800
C	2.59298300	0.79965000	1.25845600
N	-0.38768400	-2.02642500	2.51534100
N	3.81474700	2.80247600	1.78678800
N	2.94672800	1.76722900	2.15505300
O	3.07835700	0.73383200	0.14890000
H	0.93231200	-0.62897700	-0.26059700
H	1.96772300	-0.04111500	3.86628800
H	-0.81057500	-2.25252800	0.51599300
H	0.20356000	-1.67621300	4.45236700
H	4.76156200	2.43422200	1.78730900
H	3.60731400	3.05744100	0.82324200
H	2.33701500	1.98228500	2.92860200

β-CD

Cartesian atomic coordinates:

C	1.53902500	-5.23562900	0.34263200
C	-3.15469900	-4.63536800	-0.42080100
C	-5.47477400	-0.43531400	-0.15459600
C	5.07072700	-2.03258800	-0.03468700
C	-3.65638100	4.02966400	0.29162700
C	0.90425200	5.51210900	-0.13145700
C	4.82443100	2.75447300	-0.20957200
C	2.50273300	-5.19104200	-0.83934700
C	-2.22852800	-4.77083200	-1.61606100
C	-5.09762400	-1.02252900	-1.50128800
C	4.99066600	-1.35185800	-1.38894700
C	-4.36365600	3.49898800	-0.93783000
C	0.02462900	5.37289900	-1.35813100
C	4.31649400	3.42598400	-1.48074200
C	3.94127300	-5.21830200	-0.33477100
C	-1.42908400	-6.05338500	-1.49199500

C	-5.66281700	-2.42900500	-1.61485400
C	6.11542400	-0.33457800	-1.50276200
C	-5.73172700	2.95312100	-0.55501300
C	-1.23891600	6.20455100	-1.16985900
C	4.17945200	4.92596700	-1.23729200
C	1.84325800	-4.17187700	1.39231500
C	-2.36583400	-4.73196000	0.88477100
C	-5.04186400	-1.36799300	0.97924700
C	5.07970000	-0.98782900	1.08510800
C	-3.57046000	2.94097600	1.36246700
C	0.11638800	5.11673400	1.12162700
C	3.94627400	3.07215900	0.99627000
C	4.12648300	-4.12655900	0.72383400
C	-0.68022700	-6.06055800	-0.16001100
C	-5.17351600	-3.27701000	-0.43525600
C	-5.55507000	1.88780400	0.52828200
C	6.03812800	0.65812800	-0.33871600
C	-1.95017800	5.75337600	0.10333900
C	3.30773300	5.14478800	0.00483500
C	1.04804800	-4.37685100	2.67087300
C	-3.26681700	-4.79011500	2.10471000
C	-5.55997700	-0.90455300	2.32844500
C	5.32516600	-1.60164000	2.45111000
C	-3.06649000	3.48362500	2.68816500
C	0.87392700	5.40790400	2.40416200
C	4.54557900	2.58782600	2.30500300
O	0.22425700	-5.00184200	-0.15737900
O	-3.78262000	-3.36199600	-0.52732300
O	3.94054400	-2.87007000	0.09737900
O	-4.82499600	0.81513800	-0.03426100
O	-2.35009900	4.41893900	-0.09427300
O	2.01932300	4.65509700	-0.29477900
O	4.83068500	1.34894300	-0.44603500
O	3.21416300	-4.25336000	1.78139100
O	-1.60894900	-5.94084200	0.89744400
O	-5.58021800	-2.67545800	0.76807300
O	-4.87164800	2.40852700	1.63421400
O	6.13345000	-0.04574300	0.87633200
O	-1.09639400	5.87077800	1.21086000
O	3.82985100	4.48821900	1.13316400
O	2.28561100	-6.29363300	-1.68400000
O	-2.94426000	-4.83605400	-2.83214100
O	-5.63002000	-0.18220100	-2.50909900
O	5.10617000	-2.34420100	-2.39021700
O	-4.47277600	4.55871400	-1.87094900
O	0.75234900	5.81771500	-2.48517400

O	5.21877800	3.19484800	-2.53585300
O	4.89898400	-5.06403100	-1.34568300
O	-0.49446600	-6.18176900	-2.54109200
O	-5.22671000	-2.95797600	-2.86087400
O	5.98376000	0.31638900	-2.75982300
O	-6.41829100	2.46433200	-1.68014100
O	-2.05649300	6.13638800	-2.31188800
O	3.66613500	5.63839100	-2.32484300
O	1.36507300	-5.60423300	3.28834700
O	-4.15053100	-5.88992000	2.03994000
O	-6.96750600	-0.78411800	2.33282200
O	6.54564800	-2.31170100	2.49302100
O	-3.92679100	4.47855300	3.19726500
O	1.19160200	6.77895500	2.51664900
O	5.80130400	3.18187000	2.54998700
H	1.59292300	-6.22430400	0.81243500
H	-3.90788400	-5.43077700	-0.44804300
H	-6.56301300	-0.30449300	-0.12073700
H	5.99196100	-2.62579000	0.00668700
H	-4.20914600	4.88685500	0.69275100
H	1.23518200	6.55326500	-0.04319800
H	5.84328900	3.10325000	-0.00649300
H	2.33933100	-4.24549600	-1.37640200
H	-1.53252500	-3.92213400	-1.62287300
H	-4.00508100	-1.06893400	-1.58158800
H	4.02907500	-0.83147100	-1.47493200
H	-3.76155000	2.68566000	-1.36539800
H	-0.25237100	4.31772500	-1.48070600
H	3.32543700	3.01613400	-1.72052700
H	4.12958900	-6.19077200	0.13034300
H	-2.12218800	-6.90444900	-1.49488300
H	-6.75624500	-2.37305400	-1.58313800
H	7.07167300	-0.86574200	-1.44478500
H	-6.33887400	3.76675200	-0.14664300
H	-0.94646000	7.25224400	-1.04193500
H	5.17545700	5.33361500	-1.03670700
H	1.63200300	-3.18047100	0.97403900
H	-1.69610800	-3.86514900	0.95573300
H	-3.94619300	-1.41495300	1.00166000
H	4.11118800	-0.47147300	1.08534800
H	-2.90881400	2.14194900	1.00685700
H	-0.11548800	4.04486900	1.06339200
H	2.95390200	2.62627100	0.85137500
H	5.12300900	-4.18443100	1.16897800
H	-0.17053900	-7.01407500	-0.00713900
H	-5.62616100	-4.27256400	-0.43862900

H	-6.51939600	1.53539200	0.90366400
H	6.88434700	1.35030100	-0.35042100
H	-2.81574700	6.38443400	0.31800800
H	3.26276300	6.20430300	0.26588800
H	1.23945500	-3.53483100	3.34665500
H	-0.01677000	-4.40757900	2.43509100
H	-3.88462100	-3.89166500	2.14683700
H	-2.64166200	-4.83670200	3.00344300
H	-5.21936800	-1.60627600	3.09764600
H	-5.15423200	0.08377000	2.55246400
H	5.30724500	-0.80420500	3.20275600
H	4.53056500	-2.31450500	2.67878100
H	-2.95846400	2.65288400	3.39484100
H	-2.09323000	3.95329600	2.53932100
H	0.26848700	5.07245700	3.25457100
H	1.81831200	4.86212200	2.40242300
H	3.84144100	2.80468100	3.11736000
H	4.70994800	1.51095800	2.25664200
H	1.42349100	-6.19053300	-2.11144800
H	-3.49634000	-4.04430500	-2.90534500
H	-5.40557900	-0.55705000	-3.36732300
H	5.06749200	-1.91029500	-3.24880200
H	-5.12303100	4.30089600	-2.53536900
H	0.12754000	5.91765600	-3.21314300
H	5.22906500	2.24286500	-2.70301900
H	4.76516100	-4.21443000	-1.79162800
H	-0.95037400	-5.98060600	-3.36628000
H	-5.87374100	-3.58459200	-3.19682900
H	6.85372000	0.52686000	-3.10968000
H	-6.01195000	1.63410700	-1.98020900
H	-2.74554000	5.46227000	-2.19669100
H	2.71899700	5.45652100	-2.42476700
H	2.31908700	-5.61624700	3.42083300
H	-3.61206400	-6.68809100	2.02227400
H	-7.33817500	-1.66142000	2.19434800
H	7.25361000	-1.68367500	2.31709300
H	-4.79310600	4.07623400	3.31733900
H	0.36225700	7.26790900	2.51266400
H	5.66926000	4.13598200	2.54547500

ISO···β-CD complex in configuration A

Cartesian atomic coordinates:

C	1.61046700	1.30987700	-0.35487300
C	-3.01044600	-0.05397700	-0.84603100
C	-6.51882500	-2.09080900	1.74688100
C	4.23724000	0.10493200	3.42639700
C	-6.14586900	-4.99878000	5.34557500
C	-1.74809000	-5.06358600	7.34571900
C	2.55800300	-3.28537500	6.34600000
C	1.82993900	2.34400600	0.73459600
C	-2.68183000	1.43244100	-0.75216400
C	-6.07874500	-0.69854400	2.19360200
C	3.58008300	0.56609200	4.72125800
C	-6.84091200	-3.65275400	5.51800600
C	-2.61224600	-3.81425700	7.37771300
C	1.65601800	-2.95163500	7.51932700
C	3.31062400	2.53331800	1.00199000
C	-1.60067200	1.80784300	-1.74971000
C	-6.07708500	0.22893800	0.98223200
C	4.19288800	-0.12153800	5.92263800
C	-8.02868800	-3.47729200	4.58100700
C	-3.97335500	-4.14830400	7.97588700
C	1.25600500	-4.23076100	8.22662400
C	2.35504200	0.01359300	-0.03375400
C	-1.72436300	-0.85875300	-0.67447400
C	-5.53971500	-2.57464300	0.68091500
C	4.25439400	-1.42012200	3.35486500
C	-5.97056500	-5.37984200	3.87040600
C	-2.47150800	-6.17820600	6.58841700
C	1.85585300	-4.27531600	5.42031700
C	3.96862700	1.17693700	1.24319900
C	-0.40270600	0.86828900	-1.62163100
C	-5.28408700	-0.40808500	-0.17501500
C	-7.56128500	-3.78412500	3.16554400
C	4.18004200	-1.63359200	5.71703400
C	-4.59455700	-5.32156800	7.20161800
C	0.65469600	-5.23001900	7.23369200
C	2.23492900	-1.04290700	-1.11012100
C	-1.89853700	-2.36362200	-0.77369700
C	-5.67657300	-4.03782600	0.28180800
C	5.04544500	-2.01515100	2.20754300
C	-5.68240200	-6.86020500	3.68848000
C	-1.70878500	-7.48781700	6.59789700
C	2.70302900	-4.70043800	4.23887700
O	0.20570200	1.11797600	-0.38674100

O	-3.92900300	-0.37045200	0.19322000
O	3.41180000	0.65769800	2.41874100
O	-6.49600600	-2.91601400	2.89083300
O	-4.83088500	-4.92707800	5.87438800
O	-0.54302900	-4.72980700	6.68018900
O	2.85006400	-2.07236600	5.67417600
O	3.74030200	0.32307200	0.15660600
O	-0.82627400	-0.46635900	-1.71244900
O	-5.69293200	-1.72441200	-0.45139700
O	-7.14365300	-5.12327000	3.09975400
O	4.85805900	-1.95222800	4.53466000
O	-3.73367100	-6.43175900	7.21481400
O	1.55485500	-5.45188700	6.18654600
O	1.29374200	3.59902700	0.37487200
O	-3.79759400	2.25952900	-1.02413700
O	-6.89813300	-0.21726300	3.23436600
O	3.70908500	1.95722300	4.90384500
O	-7.27196300	-3.54543800	6.87417000
O	-1.99182200	-2.75501200	8.07979500
O	2.29789700	-2.13609500	8.47429700
O	3.52658600	3.33435700	2.15156200
O	-1.14866400	3.13073900	-1.53562000
O	-5.53505900	1.50438100	1.27425100
O	3.48168700	0.17607600	7.11010200
O	-8.56788400	-2.18479400	4.70224400
O	-4.78044800	-2.99610000	7.93044300
O	0.31124500	-3.95600600	9.25240900
O	2.41654500	-0.43483600	-2.38206500
O	-0.73553600	-3.04930800	-0.36897100
O	-4.71733700	-4.75019000	1.04318400
O	4.88401500	-3.41523600	2.17044600
O	-5.63374400	-7.19598900	2.30939300
O	-1.41232500	-7.91952600	7.91082100
O	4.01812200	-5.07760800	4.60725200
H	1.96481300	1.70387800	-1.31346200
H	-3.45377400	-0.28824200	-1.82250500
H	-7.53356100	-2.05082900	1.32751000
H	5.25542600	0.51247800	3.36254100
H	-6.72987400	-5.76851200	5.86948100
H	-1.54318300	-5.39892300	8.36899800
H	3.48290600	-3.74826000	6.70650000
H	1.35645800	1.97483800	1.65479900
H	-2.29792800	1.62529400	0.25938000
H	-5.04822700	-0.78785500	2.56211100
H	2.51979100	0.28142600	4.66681600
H	-6.10957200	-2.86254500	5.30637500

H	-2.77307400	-3.48505300	6.34451600
H	0.75029100	-2.46209500	7.13341600
H	3.77598700	2.99076500	0.12114700
H	-2.00951800	1.70066800	-2.76246800
H	-7.10658100	0.39744500	0.65640800
H	5.23931700	0.19283800	6.01857800
H	-8.82018300	-4.18282200	4.84992600
H	-3.83504600	-4.47475500	9.01557600
H	2.15137700	-4.69049700	8.66225900
H	1.95002700	-0.40761000	0.89416000
H	-1.28356200	-0.62360200	0.30368600
H	-4.52450300	-2.46498400	1.07392700
H	3.21477100	-1.76222600	3.28649400
H	-5.14244500	-4.79978900	3.45607000
H	-2.62641100	-5.85909800	5.55075600
H	0.92366900	-3.84036500	5.04140600
H	5.05329800	1.27861600	1.33802200
H	0.30716500	1.01795000	-2.43681600
H	-5.44748700	0.15637300	-1.09521000
H	-8.36935400	-3.66450400	2.43905100
H	4.72216200	-2.13060800	6.52468700
H	-5.52472700	-5.64894100	7.67585600
H	0.47917400	-6.19592300	7.71693600
H	3.00249800	-1.80191900	-0.92260800
H	1.24752100	-1.50789200	-1.04001700
H	-2.08426900	-2.62964300	-1.81731800
H	-2.77170900	-2.66367100	-0.18352100
H	-6.68921600	-4.41106000	0.47686800
H	-5.47140400	-4.12354400	-0.79015600
H	4.68364100	-1.61762400	1.25802600
H	6.10155000	-1.73656300	2.32668400
H	-4.71168500	-7.10245200	4.12168300
H	-6.45112600	-7.45481600	4.19535300
H	-2.29150700	-8.24094400	6.05626000
H	-0.75617100	-7.35308600	6.08137800
H	2.18971700	-5.51097900	3.71246700
H	2.80755100	-3.86481800	3.54806900
H	0.43771100	3.45605800	-0.05803800
H	-4.42245200	2.21000800	-0.28549100
H	-6.32306500	-0.12719500	4.01064100
H	3.48762500	2.40280600	4.06987400
H	-7.86164000	-2.78093300	6.91849600
H	-1.22963600	-3.10275400	8.57029300
H	2.55063000	-1.29458300	8.06075400
H	3.03699300	4.15464100	2.02199300
H	-1.92635400	3.70024400	-1.54799000

H	-4.76439200	1.36981000	1.83768400
H	3.36074600	1.13190900	7.14576600
H	-7.99640500	-1.55170100	4.23148100
H	-5.70672200	-3.23145500	7.75819200
H	0.72584200	-3.30965900	9.83801200
H	2.26277000	-1.09971500	-3.05671700
H	-0.66425600	-2.99589600	0.59705100
H	-5.01428800	-5.64984000	1.23815900
H	5.09342000	-3.77735100	3.04256400
H	-6.53800900	-7.15127800	1.98175900
H	-2.24670300	-8.03170000	8.37745700
H	3.97938900	-5.92656400	5.05655900
C	-3.26882000	-2.49005000	3.61074600
C	-1.44726900	-1.17822800	4.44207800
C	-2.38930100	-3.03015800	2.67919900
C	-0.65541100	-1.75504400	3.45246100
C	-2.79142100	-1.53719900	4.50618400
C	-3.75639200	-0.91157300	5.47366500
N	-1.10861800	-2.65617100	2.57903100
N	-4.06264000	-0.12322600	7.71661600
N	-3.26952000	-0.66040600	6.69198600
O	-4.90538400	-0.62098000	5.12942200
H	-4.31018600	-2.79188600	3.63191000
H	-1.02792900	-0.43684800	5.11183900
H	-2.73888100	-3.78066300	1.97468000
H	0.38791900	-1.46851700	3.35456500
H	-4.74304200	0.48357500	7.26864200
H	-4.56803200	-0.90973400	8.12802400
H	-2.43152900	-1.12572900	7.02977900

ISO···β-CD complex in configuration B

Cartesian atomic coordinates:

C	5.88715700	0.21072900	-0.52644400
C	3.93437400	0.19821400	3.81952700
C	-0.72346400	-0.15589000	4.99168600
C	3.14354200	-0.70214200	-4.36240600
C	-4.57728100	1.19887500	2.70090700
C	-4.97921200	0.83535600	-1.96243100
C	-1.65722300	-1.07860600	-4.83902000
C	5.85212800	-1.11308800	-1.26137100
C	4.59879500	-1.06574100	3.27700100
C	-0.11847600	-1.07527900	3.93371200
C	2.39152800	-2.00063200	-4.16195700
C	-4.06904700	-0.20074500	3.02330600
C	-5.58296200	-0.05607900	-0.89566500
C	-2.24647900	-1.92494400	-3.72005100
C	6.13977900	-0.92878700	-2.74193900
C	6.05625900	-0.79457000	2.90876800
C	1.10150600	-1.78065700	4.52248800
C	1.54095700	-2.29079800	-5.39195000
C	-3.96694800	-0.36276900	4.53121700
C	-6.46536100	0.74648900	0.04477900
C	-3.76779800	-1.93968400	-3.81501200
C	4.96311200	1.23097000	-1.19923400
C	4.10254400	1.28486400	2.75184500
C	0.33721800	0.81590200	5.50431000
C	2.16386700	0.43226200	-4.67052400
C	-3.68668500	2.24446900	3.37974000
C	-4.31809900	2.07762900	-1.36223000
C	-2.28924600	0.31164400	-4.83816900
C	5.15920400	0.10790200	-3.29663100
C	6.10164000	0.36473800	1.91762200
C	2.08278000	-0.72882900	5.06007200
C	-2.99718700	0.70303300	5.04567500
C	0.63058000	-1.09473600	-5.67092300
C	-5.62868600	1.89415300	0.61551800
C	-4.28473100	-0.49783800	-3.85201000
C	5.12486800	2.62991700	-0.62279200
C	3.44133400	2.62580200	3.04059000
C	-0.15158700	1.68505200	6.64669100
C	2.87164500	1.72954700	-5.01408400
C	-4.25441200	3.64648200	3.28965600
C	-4.01462600	3.11629700	-2.42867900

C	-1.87511000	1.18452100	-6.00495700
O	5.41887500	-0.08350500	0.78083500
O	2.53824000	0.02186100	3.96411900
O	3.85702700	-0.43210500	-3.17287200
O	-1.76291400	0.59809400	4.38347000
O	-4.49944000	1.38098000	1.29670600
O	-3.96013700	0.11286000	-2.63296600
O	-0.26631000	-0.98947700	-4.58947300
O	5.26000700	1.31005500	-2.59429200
O	5.48723100	1.49465600	2.48236700
O	1.45935900	0.09936300	6.00987800
O	-3.54194600	1.96320900	4.77575100
O	1.37383900	0.08253100	-5.81552400
O	-5.18297600	2.71824800	-0.41879100
O	-3.71109700	0.19789300	-4.92453100
O	6.78353100	-1.98262100	-0.63810500
O	4.52478500	-2.13312500	4.21490700
O	-1.04757000	-1.99428100	3.40545700
O	3.33106700	-3.03919000	-3.94376300
O	-4.91108800	-1.20728300	2.48199700
O	-6.32820800	-1.08402400	-1.54616700
O	-1.68496900	-3.21993200	-3.82383900
O	6.07020500	-2.15984800	-3.42083700
O	6.65857300	-1.96263500	2.40458000
O	1.70205400	-2.56427100	3.51928200
O	0.82005700	-3.48801000	-5.24258800
O	-3.47113100	-1.64437900	4.87501000
O	-7.03921700	-0.07474200	1.03278000
O	-4.29233400	-2.65654100	-2.71623200
O	3.98412200	3.42694900	-0.84454200
O	2.90778700	3.18233700	1.84939900
O	0.85294100	2.57410900	7.07428900
O	3.76644200	1.55982200	-6.09720500
O	-3.37182100	4.59555900	3.83619400
O	-5.19010900	3.54478200	-3.08309400
O	-2.43601000	2.47243100	-5.88421300
H	6.91845100	0.58702400	-0.48875700
H	4.39092900	0.50051300	4.77232400
H	-1.12067300	-0.75630600	5.82233100
H	3.83661400	-0.82167400	-5.20379200
H	-5.61588000	1.29826900	3.04622000
H	-5.75722700	1.15103100	-2.66752900
H	-1.84294100	-1.58197900	-5.79787600
H	4.83813500	-1.52375300	-1.15791000
H	4.05453600	-1.35575200	2.36932100
H	0.22806200	-0.44381500	3.11047300

H	1.73687400	-1.90078800	-3.28541400
H	-3.06581200	-0.32211900	2.60680800
H	-4.76209500	-0.50956700	-0.32537700
H	-1.97042400	-1.46228500	-2.76285600
H	7.15840300	-0.55242200	-2.87407500
H	6.60638500	-0.50824200	3.81041900
H	0.77853500	-2.40733700	5.36614600
H	2.20640300	-2.42722700	-6.25027200
H	-4.94256300	-0.19857700	5.00448100
H	-7.29610700	1.18600400	-0.51607700
H	-4.06020600	-2.42874200	-4.75225500
H	3.92521700	0.90328600	-1.06119100
H	3.62854300	0.90457800	1.84357900
H	0.65180300	1.45482600	4.67003800
H	1.51094200	0.59052300	-3.80417800
H	-2.69994200	2.22012600	2.90436500
H	-3.38808600	1.77798100	-0.86783600
H	-2.01749200	0.81544500	-3.90315400
H	5.37516000	0.34375800	-4.34186700
H	7.12825400	0.65046400	1.67433400
H	2.91586800	-1.20003500	5.58269500
H	-2.87164500	0.63412700	6.13035800
H	0.08577300	-1.23263300	-6.60886000
H	-6.22791700	2.51899900	1.28437400
H	-5.36513000	-0.48006000	-4.01763000
H	5.33443800	2.55311300	0.44736800
H	5.98876100	3.09109400	-1.11414300
H	4.18363200	3.32612100	3.42512600
H	2.65547200	2.49620400	3.79064100
H	-0.99541000	2.29041700	6.30955600
H	-0.48305600	1.03805700	7.47163200
H	2.11953900	2.49361400	-5.23721600
H	3.46026400	2.06311600	-4.15730900
H	-4.39617000	3.90977200	2.23904300
H	-5.22938400	3.66939400	3.79669900
H	-3.48837400	3.95756600	-1.96596100
H	-3.36741500	2.67797700	-3.19044400
H	-0.79030500	1.30210200	-6.01066300
H	-2.18475800	0.70145000	-6.94209000
H	6.92432800	-2.72774000	-1.23455300
H	5.20310600	-2.77018500	3.95237900
H	-1.82340300	-2.02716300	3.98189700
H	2.88683700	-3.87802000	-4.11569300
H	-4.38090000	-1.66081100	1.80513700
H	-6.93938400	-1.45426100	-0.89664000
H	-1.94355500	-3.74790000	-3.04858400

H	5.14430200	-2.45124700	-3.47729400
H	6.52518600	-2.01083000	1.44229200
H	2.60833200	-2.75983000	3.79524100
H	0.04595000	-3.34234500	-4.66950700
H	-4.08248500	-2.29177200	4.50333400
H	-6.33420700	-0.50321700	1.55225400
H	-5.08012700	-2.20832800	-2.36393400
H	3.57165400	3.57345200	0.01675500
H	2.21950800	2.56694000	1.53582600
H	1.63579100	2.04515300	7.25961400
H	3.24811400	1.31240200	-6.86904700
H	-3.16993100	4.32055200	4.73619700
H	-5.74424400	3.98260100	-2.42967800
H	-3.39273500	2.37191800	-5.83360400
C	-1.25992900	0.36092600	0.75965200
C	0.51583600	-1.23175100	0.56884200
C	-0.29223400	1.32519800	1.02064700
C	1.40378100	-0.19252800	0.81896400
C	-0.84451200	-0.94511800	0.52172800
C	-1.87124300	-1.99544500	0.21568500
N	1.01860000	1.06496400	1.04179100
N	-2.29055200	-3.97525600	-1.09691600
N	-1.43415200	-2.99202900	-0.58847000
O	-3.02468100	-1.94570600	0.62082100
H	-2.31315100	0.61896000	0.74021600
H	0.89392600	-2.24214100	0.47513200
H	-0.57996700	2.35366000	1.21631400
H	2.46962500	-0.39430900	0.85124500
H	-2.41062500	-4.70421200	-0.40006100
H	-3.19772000	-3.54484600	-1.29219900
H	-0.55818200	-2.89017000	-1.07999500

ISO···β-CD complex in configuration C

Cartesian atomic coordinates:

C	2.46989500	4.03827700	-0.45649800
C	0.28102900	2.85491400	-4.50158300
C	-3.04244200	-0.43356200	-5.59172900
C	3.58636800	2.23958900	3.89344900
C	-3.22995200	-3.45950200	-1.90764400
C	-1.48817900	-3.75116200	2.54691600
C	1.88762600	-1.81003700	5.32589800
C	1.36988400	3.81611700	0.57543300
C	-0.10763300	3.90963500	-3.48016700
C	-3.44337300	0.98911600	-5.24280300
C	2.46654600	2.26901700	4.92468000
C	-4.49646100	-2.67642200	-2.22909000
C	-2.44449000	-2.60156500	2.26072100
C	0.38725600	-1.82839300	5.53182400
C	1.76030400	4.50714600	1.87924600
C	1.08008800	4.82947400	-3.25515000
C	-2.74339900	2.00422300	-6.12806500
C	2.83866800	1.53642200	6.20806300
C	-4.91010900	-2.89079300	-3.68182100
C	-3.71133100	-3.15627100	1.62280000
C	-0.16062400	-3.24418100	5.47404000
C	3.79777500	3.52405900	0.09620800
C	1.51261800	2.09666700	-3.99885000
C	-1.51602100	-0.58789400	-5.70649700
C	4.12528100	0.81748700	3.70740900
C	-2.14859900	-2.99795800	-2.88314000
C	-1.19912000	-4.53650300	1.27071300
C	2.24725200	-2.55733800	4.04072500
C	3.14955400	4.02002100	2.32243300
C	2.31832600	4.00547200	-2.86516600
C	-1.23896600	1.77206900	-6.04365900
C	-3.73387100	-2.56516900	-4.60250300
C	3.31039900	0.14009800	5.82258600
C	-3.34377100	-4.01822700	0.40718800
C	0.28396500	-3.90082400	4.15964100
C	4.97761700	3.75734100	-0.82679000
C	1.97911400	0.98822100	-4.93173200
C	-1.07012900	-1.85021500	-6.44292300
C	5.39901700	0.77727400	2.87872300
C	-0.79993700	-3.67072500	-2.71412000
C	-0.31083200	-5.74382300	1.49917100

C	3.74056800	-2.72519600	3.83070000
O	2.10933900	3.34749800	-1.63821200
O	-0.83950200	2.02304300	-4.71095700
O	3.07075900	2.65995400	2.63901400
O	-3.56134100	-1.18453900	-4.50148400
O	-2.81115800	-3.17652100	-0.58706900
O	-0.27742800	-3.21409500	3.05582400
O	2.21130900	-0.43967300	5.18414600
O	4.09440300	4.23127200	1.30192000
O	2.57170700	3.05375100	-3.84864200
O	-0.92403000	0.48090800	-6.45460700
O	-2.58825700	-3.27476200	-4.21319100
O	4.42355600	0.23759900	4.97354700
O	-2.42469900	-5.03078000	0.73545900
O	1.67879000	-3.86706800	4.03991100
O	0.10459800	4.25342400	0.11254800
O	-1.21761000	4.63071700	-3.98203600
O	-4.84856800	1.08376800	-5.35349200
O	2.14119900	3.62958100	5.21168800
O	-5.54483500	-3.07216700	-1.35525200
O	-2.72847900	-1.91938100	3.46631000
O	0.11678400	-1.19824100	6.77173700
O	0.78018300	4.25100700	2.85382100
O	0.70549300	5.76180800	-2.23936900
O	-3.11019900	3.31522600	-5.77254200
O	1.74373900	1.51309700	7.09241800
O	-6.04542900	-2.11676800	-3.99271900
O	-4.55218600	-2.09149000	1.26078600
O	-1.55565600	-3.24177200	5.64540900
O	6.16697900	3.26802800	-0.25609300
O	1.32380300	-0.19387500	-4.53361200
O	0.26089000	-2.19852000	-6.12113600
O	5.87171600	-0.53181100	2.68154700
O	0.20016400	-3.00718600	-3.46235300
O	-0.86332500	-6.63042600	2.45042800
O	4.06847900	-2.81972900	2.45163300
H	2.56767300	5.11459900	-0.65207500
H	0.54972100	3.36025700	-5.43958500
H	-3.54257200	-0.73324100	-6.52385900
H	4.40245600	2.90209900	4.21376100
H	-3.39984100	-4.53802700	-2.03216900
H	-1.94578300	-4.42017000	3.28513900
H	2.39553500	-2.24294000	6.19826300
H	1.28208000	2.74068600	0.76799200
H	-0.36197700	3.41709000	-2.53196700
H	-3.13387300	1.16835400	-4.20354100

H	1.59406600	1.77602900	4.47998500
H	-4.27436800	-1.61236800	-2.07084100
H	-1.98307200	-1.91455400	1.54588300
H	-0.06572700	-1.24808900	4.71378100
H	1.83722900	5.58831500	1.69355400
H	1.29351600	5.36019400	-4.18910600
H	-3.05305600	1.85387800	-7.16679600
H	3.65431200	2.06280100	6.71164000
H	-5.18850800	-3.93932700	-3.82035300
H	-4.20748100	-3.81248100	2.35212700
H	0.25679300	-3.83087700	6.29876400
H	3.71323300	2.45207500	0.30697000
H	1.30647500	1.63664300	-3.02894100
H	-1.09922500	-0.58946200	-4.69219600
H	3.34967800	0.22403300	3.20453200
H	-2.01052800	-1.91705200	-2.75871500
H	-0.71793100	-3.86357500	0.54993200
H	1.84183500	-1.98050100	3.20074200
H	3.49901200	4.59896500	3.18127300
H	3.20693000	4.64470300	-2.81387700
H	-0.71052200	2.44504400	-6.72429200
H	-3.94623000	-2.85975400	-5.63520100
H	3.62363200	-0.44402800	6.69221400
H	-4.22851100	-4.53573100	0.03219500
H	-0.00530400	-4.95509500	4.13628600
H	4.82267800	3.21487300	-1.76172700
H	5.05785600	4.83260700	-1.04486500
H	3.06554900	0.89613300	-4.82808600
H	1.74948100	1.23588900	-5.97553300
H	-1.73845600	-2.68342900	-6.22858700
H	-1.10461000	-1.63659400	-7.51374400
H	5.21776300	1.29929500	1.93100200
H	6.18756400	1.30790600	3.41896200
H	-0.54537800	-3.68756200	-1.65080600
H	-0.85942000	-4.69890100	-3.07879700
H	-0.14627200	-6.24671300	0.53949600
H	0.65486800	-5.41683400	1.89076300
H	4.28757300	-1.86075200	4.20612900
H	4.07538900	-3.61266400	4.37838900
H	0.21980100	4.94427500	-0.55435500
H	-1.45232900	5.31158100	-3.34281800
H	-5.07288900	2.01975200	-5.29512800
H	1.61253400	3.62257900	6.02083000
H	-6.36707500	-2.73157400	-1.73032600
H	-2.48365700	-0.98710500	3.34343800
H	-0.79803800	-1.40056800	7.00191300

H	1.20225300	4.18539700	3.72697200
H	1.10691400	6.61891300	-2.40344900
H	-2.54754300	3.62195500	-5.04320200
H	1.15740100	0.77648900	6.85012300
H	-5.74443200	-1.21299800	-4.17216800
H	-5.07284800	-2.35270100	0.48894600
H	-1.97349100	-2.72708100	4.92662100
H	6.24999400	3.66659800	0.61639300
H	1.14725700	-0.76241600	-5.30132800
H	0.24508900	-2.66202400	-5.26285200
H	5.22417700	-1.07012300	2.20817100
H	0.47048500	-2.21119400	-2.96157500
H	-1.73602000	-6.88729000	2.13484600
H	3.72448300	-3.64711800	2.10403700
C	-0.97476900	0.89822500	-0.65221100
C	0.05908700	-0.60846900	0.89430700
C	-0.44341000	0.12416500	-1.67978900
C	0.55474400	-1.30539900	-0.19941200
C	-0.73006300	0.51444500	0.66398500
C	-1.32390200	1.21408100	1.85267600
N	0.30145400	-0.96206300	-1.46452900
N	-2.08570600	3.30798800	2.72946500
N	-1.51793100	2.52551000	1.71434400
O	-1.62128300	0.59140600	2.87605900
H	-1.60407200	1.74897600	-0.88600900
H	0.23886500	-0.95315100	1.90634900
H	-0.62921000	0.37924800	-2.72113700
H	1.17243900	-2.18702600	-0.05284900
H	-1.31060200	3.78274300	3.19188000
H	-2.50253700	2.65608700	3.38934300
H	-1.16553300	3.05639300	0.92331400

ISO···β-CD complex in configuration D

Cartesian atomic coordinates:

C	-3.39174300	0.54920600	-4.41541800
C	-4.85159500	-1.43054900	-0.38326400
C	-2.92386300	-2.84122900	3.72433400
C	1.01471200	1.65858500	-5.52516400
C	1.30270900	-1.16693400	5.12024300
C	4.73463200	0.78995200	2.35618400
C	4.60181000	2.07033600	-2.26622600
C	-3.11004400	2.04689700	-4.51609900
C	-4.56336000	0.06040800	-0.41857900
C	-3.99518700	-1.78430700	3.51455700
C	1.62030600	2.98400500	-5.09868800
C	0.06409800	-0.28821300	5.07323500
C	3.86361400	1.54658100	3.33797100
C	4.26696100	3.08896100	-1.19723500
C	-2.50909600	2.37010900	-5.88303300
C	-5.44949600	0.69080900	-1.48348700
C	-5.16322100	-2.33468100	2.72318600
C	3.13640400	2.96988900	-5.23665700
C	-0.98266400	-0.90134000	5.99109700
C	3.97328100	0.89496900	4.71123100
C	5.41186700	3.17408700	-0.19453600
C	-2.16692000	-0.29098500	-4.74441500
C	-4.57198100	-2.03378000	-1.76398700
C	-2.57541200	-3.55926300	2.41939200
C	1.68366700	0.53212200	-4.73018500
C	0.94131500	-2.60649700	4.73364300
C	4.33492000	-0.68628600	2.32196800
C	4.93952600	0.71970900	-1.63108600
C	-1.34039200	1.41639300	-6.14980700
C	-5.12499100	0.02230900	-2.81944400
C	-4.66874100	-2.98769100	1.43222300
C	-1.30238300	-2.30435700	5.47643500
C	3.70501000	1.75933200	-4.49408300
C	3.58988200	-0.58080600	4.59056700
C	5.70141500	1.78041100	0.36071100
C	-2.42333800	-1.80315200	-4.70869300
C	-4.92386800	-3.50485600	-1.83921000
C	-1.75330000	-4.81200400	2.67056900
C	1.20210200	-0.87090000	-5.08724100
C	2.09787100	-3.56429800	4.94975100
C	5.28961200	-1.52745600	1.49487000
C	5.43829300	-0.29555100	-2.64355700
O	-3.77835800	0.26049200	-3.08025400

O	-4.02755800	-2.03628900	0.60458900
O	-0.35960100	1.73199400	-5.19286700
O	-1.75080200	-2.16681600	4.15961100
O	2.23521200	-0.64747600	4.19542700
O	4.56046100	1.37909800	1.07856900
O	3.47069000	1.95359000	-3.11091800
O	-1.72895100	0.06846800	-6.05652200
O	-5.38613000	-1.36310400	-2.73218800
O	-3.75809000	-3.99802500	1.74884000
O	-0.14025600	-3.09201800	5.53544100
O	3.09860500	0.57490900	-4.92378300
O	4.39094100	-1.23154900	3.64484500
O	5.99361600	0.88469400	-0.68034500
O	-4.30433700	2.77222200	-4.34241000
O	-4.74176000	0.69778500	0.83746400
O	-4.52051100	-1.30278300	4.73404400
O	1.03044300	4.00681000	-5.87434500
O	0.34713700	1.04216900	5.46198000
O	4.31041000	2.89107300	3.36777200
O	4.04916400	4.33639800	-1.82942400
O	-2.09222400	3.70155100	-6.01564800
O	-5.20943600	2.07535600	-1.60478500
O	-6.06934000	-1.28587600	2.40695800
O	3.69425100	4.17939200	-4.78182300
O	-2.17623400	-0.13113600	6.00301100
O	3.19937600	1.57320300	5.66935200
O	5.12753100	4.11313300	0.81175400
O	-1.75842200	-2.44884000	-3.63923100
O	-6.27093700	-3.74564200	-1.47523300
O	-2.47288300	-5.75435300	3.43424600
O	0.97551700	-1.68620800	-3.94178900
O	2.53893700	-3.55343900	6.29205400
O	6.61014500	-1.44260400	1.98599800
O	6.56342000	0.18633200	-3.35014300
H	-4.20228400	0.28776400	-5.10891600
H	-5.90742200	-1.58836700	-0.13408800
H	-3.25366200	-3.57373600	4.46987700
H	1.14730900	1.51039100	-6.60490800
H	1.72005300	-1.15239700	6.13452900
H	5.78229300	0.86981800	2.66787500
H	5.46518900	2.43091700	-2.83751700
H	-2.37784900	2.30642100	-3.73972500
H	-3.52627900	0.23013800	-0.71849000
H	-3.54649100	-0.96373600	2.93807200
H	1.37061000	3.12979900	-4.03691300
H	-0.32731100	-0.29333000	4.05188400

H	2.81834700	1.50566700	3.00068500
H	3.35745700	2.77307000	-0.66835300
H	-3.27683000	2.19502100	-6.64339600
H	-6.50544500	0.50394900	-1.24834900
H	-5.67515000	-3.10437200	3.31304400
H	3.39817500	2.88125800	-6.29526000
H	-0.58866100	-0.98775500	7.00969400
H	5.01215900	0.95754000	5.04961500
H	6.30601600	3.52386500	-0.72095600
H	-1.37913700	-0.06260800	-4.01503000
H	-3.51235200	-1.91026100	-2.01683300
H	-2.01481100	-2.87220300	1.77512800
H	1.45976200	0.72371400	-3.67533500
H	0.65203100	-2.61281000	3.67520600
H	3.31620600	-0.77500700	1.92484500
H	4.04393500	0.32634600	-1.13120300
H	-0.95051000	1.55785500	-7.16111200
H	-5.76070900	0.40850700	-3.61928400
H	-5.49119400	-3.46369500	0.89105100
H	-2.04957800	-2.80117500	6.09950900
H	4.77529900	1.64296100	-4.68245400
H	3.73818900	-1.11059800	5.53545800
H	6.58014400	1.78917100	1.00935100
H	-3.48976500	-2.00125600	-4.57185500
H	-2.11876000	-2.22027300	-5.67462300
H	-4.29821800	-4.06257500	-1.13938500
H	-4.71108300	-3.86572600	-2.85059900
H	-1.44107200	-5.23380600	1.70926700
H	-0.86452600	-4.54749000	3.24739700
H	0.28681900	-0.80859900	-5.67991000
H	1.97337500	-1.35119000	-5.69270900
H	1.78909700	-4.56912900	4.64150800
H	2.94339600	-3.25356600	4.33274800
H	4.93303900	-2.56353800	1.49070900
H	5.31010000	-1.15527700	0.47061100
H	5.67207400	-1.22856300	-2.11859500
H	4.65733700	-0.49786900	-3.37837900
H	-4.54382400	2.74617800	-3.40600900
H	-5.29975100	0.13600100	1.40334200
H	-3.84399500	-0.77910800	5.18653200
H	1.54604600	4.81002500	-5.73633000
H	0.14666700	1.60282200	4.69138200
H	3.92256000	3.31212800	4.14412000
H	4.05971000	5.01701600	-1.14596000
H	-1.18721100	3.78501200	-5.68250900
H	-5.21959600	2.45458700	-0.71876900

H	-6.32036200	-0.88252800	3.24812400
H	3.64941900	4.21695400	-3.81171900
H	-1.99106000	0.67697900	6.49448600
H	2.25481900	1.38857800	5.52869600
H	4.69122500	3.67828900	1.56351300
H	-0.80141100	-2.39927600	-3.80589300
H	-6.82939400	-3.27046000	-2.09887700
H	-3.26044800	-5.98939900	2.93299300
H	0.83103900	-1.11909200	-3.16069600
H	1.81610400	-3.87469000	6.84013200
H	6.60310600	-1.77275000	2.89024300
H	7.26827500	0.33364800	-2.71134200
C	0.49650800	1.42141800	0.19448900
C	-1.21576600	-0.25523900	0.27661600
C	0.88994200	0.88308700	-1.02382100
C	-0.74380400	-0.72677800	-0.94502800
C	-0.59226600	0.85059700	0.84578200
C	-1.04654900	1.42466500	2.15457800
N	0.28427200	-0.16798300	-1.58663100
N	-2.93047300	1.75599100	3.60657500
N	-2.36237900	1.30834900	2.40794600
O	-0.25370400	1.94884500	2.93384200
H	1.01237600	2.26933900	0.62838900
H	-2.04979900	-0.75437800	0.75890600
H	1.71893200	1.31359300	-1.58019900
H	-1.21392400	-1.57355900	-1.43854800
H	-2.58326000	1.16509700	4.35945600
H	-2.61227600	2.70626600	3.78157200
H	-3.04550400	1.04805500	1.69642200

ISO···β-CD complex in configuration E

Cartesian atomic coordinates:

C	-4.85741400	-1.00802600	-1.76836200
C	-1.92355600	1.29474900	-4.84529500
C	2.76081600	2.06678400	-4.64001300
C	-3.99528700	-3.29165000	2.43098300
C	4.43941800	2.77091500	-0.24013600
C	2.81876000	1.20223500	3.94783200
C	-0.10992400	-2.48381700	4.94684600
C	-4.78140200	-2.51949700	-1.69393300
C	-2.41657400	-0.10730300	-5.15700000
C	2.23101300	0.66338800	-4.86562500
C	-2.84944600	-4.28584100	2.27769000
C	4.76375800	1.46292500	-0.94575200
C	4.15757300	0.91065700	3.29883900
C	1.18428000	-2.75820000	4.20367300
C	-5.70948400	-3.01806900	-0.59825900
C	-3.93662500	-0.16766200	-5.09312100
C	1.34293500	0.59011700	-6.09467500
C	-2.43177700	-4.83762300	3.63624400
C	5.42054700	1.72852500	-2.30026000
C	4.90011300	2.19743300	2.96339200
C	2.37591900	-2.18072200	4.95341100
C	-4.50936000	-0.41895100	-0.40334300
C	-2.47405800	1.71281100	-3.48092400
C	1.62667500	3.09635200	-4.68725200
C	-3.74688300	-2.22985500	3.50344300
C	3.62464700	3.69419300	-1.14424200
C	2.03178400	2.17611500	3.07513800
C	-0.23852300	-0.98707400	5.21813000
C	-5.41001900	-2.29532300	0.72403100
C	-4.40184400	0.33559800	-3.72797000
C	0.21968700	1.61310200	-5.92241700
C	4.55251300	2.71316700	-3.08928700
C	-2.21104800	-3.67205100	4.59941700
C	3.99274200	3.09572700	2.11557600
C	2.10523900	-0.70140700	5.25756900
C	-4.51105800	1.09866700	-0.39266600
C	-2.11735000	3.13643400	-3.08873700
C	2.11795600	4.52637900	-4.79727900
C	-4.98155700	-1.40448500	3.82107000
C	3.39546200	5.06288100	-0.53376000
C	0.74499900	2.63742200	3.72442300
C	-1.50769800	-0.60101000	5.97211900
O	-3.94842400	-0.58589400	-2.77042900

O	-0.50677500	1.28441700	-4.76430900
O	-4.13281700	-2.68898200	1.15031800
O	3.34168600	2.04220200	-3.34666600
O	3.66685700	2.44972200	0.90507800
O	2.06890100	-0.00057600	4.03572800
O	-1.15095900	-2.91331300	4.08506400
O	-5.46744500	-0.90734500	0.54266900
O	-3.89925700	1.62539200	-3.49045800
O	0.77468500	2.89888400	-5.81849300
O	4.32599500	3.90002600	-2.37269400
O	-3.36734200	-2.86789400	4.72939600
O	2.80252000	3.35011700	2.81401600
O	0.89864700	-0.54167300	5.95467600
O	-5.14670900	-3.04263100	-2.95889600
O	-1.95307300	-0.49078100	-6.44596000
O	3.34007400	-0.20932100	-4.95030100
O	-3.25083100	-5.34897600	1.44796700
O	5.60360800	0.68323600	-0.11440000
O	4.92892400	0.10301200	4.18350800
O	1.28242000	-4.14889000	3.98674800
O	-5.49172000	-4.41772900	-0.49419500
O	-4.39289400	-1.46689100	-5.37772400
O	0.88966500	-0.73647500	-6.21770500
O	-1.28513500	-5.63885600	3.58821600
O	5.63405500	0.55869900	-3.04257900
O	6.13181400	1.90576900	2.35291300
O	3.53009900	-2.34969000	4.15502400
O	-5.58220000	1.63681700	-1.14022200
O	-2.41588700	3.39112100	-1.73724800
O	1.04476400	5.43677100	-4.72123900
O	-6.08658200	-2.21632400	4.16544400
O	4.60735600	5.69340000	-0.17284400
O	-0.07584300	3.32916900	2.80778600
O	-1.90613000	-1.55224700	6.93665500
H	-5.87548700	-0.70136500	-2.02954900
H	-2.25805200	2.00065500	-5.61755700
H	3.52348800	2.28891400	-5.39864100
H	-4.90359600	-3.85160600	2.68369400
H	5.37097400	3.27191900	0.04936700
H	2.97114700	1.63251500	4.94694700
H	-0.13565100	-3.03751400	5.89108500
H	-3.75342300	-2.81501100	-1.44669500
H	-1.99883100	-0.78600200	-4.40172500
H	1.60923800	0.38928100	-4.00579400
H	-1.99399600	-3.75162400	1.84017900
H	3.82307600	0.92990100	-1.10591800

H	3.96843400	0.35756700	2.36758100
H	1.12745700	-2.22875600	3.24664900
H	-6.74346000	-2.80403700	-0.89163300
H	-4.34853800	0.49529300	-5.86089800
H	1.91746200	0.87423700	-6.98544000
H	-3.24608800	-5.46042400	4.01918500
H	6.40103200	2.18331500	-2.13064600
H	5.12810600	2.73355800	3.89029600
H	2.49229600	-2.70806400	5.90791300
H	-3.51104600	-0.76025000	-0.10251800
H	-2.07908400	1.02211600	-2.72518100
H	1.03962900	2.99539900	-3.76795300
H	-2.94087300	-1.56676200	3.16606200
H	2.65311000	3.22403300	-1.34284900
H	1.79755900	1.68721700	2.12546900
H	-0.25596600	-0.46676600	4.25094000
H	-6.16032100	-2.53486800	1.48520100
H	-5.48968700	0.42315700	-3.69094300
H	-0.43560900	1.63157100	-6.79634600
H	5.03939800	3.00126300	-4.02472500
H	-1.97495800	-4.04175300	5.60019700
H	4.46450900	4.06380300	1.92335500
H	2.88565700	-0.29440100	5.90519200
H	-4.57912800	1.43004700	0.64886200
H	-3.55691400	1.44139000	-0.79881000
H	-2.71412700	3.82696600	-3.69022600
H	-1.06029700	3.31970600	-3.31294400
H	2.79183800	4.74855500	-3.96905900
H	2.66865500	4.63749000	-5.74132500
H	-4.73776900	-0.70761700	4.63208800
H	-5.27232800	-0.82167000	2.94579900
H	2.82144700	5.67473300	-1.23853600
H	2.80998500	4.95199300	0.38148500
H	0.18153900	1.76786500	4.06682300
H	0.98442100	3.25900200	4.59649000
H	-1.31502200	0.34990100	6.47521000
H	-2.30689300	-0.44465300	5.24149200
H	-5.17109400	-4.00325000	-2.88790000
H	-2.48025400	-1.25381100	-6.71803300
H	3.00999000	-1.07177900	-5.22927600
H	-3.69484300	-4.97376100	0.67537700
H	5.18431200	-0.18271600	-0.01693900
H	5.85079300	0.17745100	3.90287500
H	1.91947600	-4.29921900	3.27023200
H	-6.23954200	-4.84281500	-0.06479200
H	-4.44330400	-1.98458800	-4.55637700

H	-0.06682400	-0.75181900	-6.38145700
H	-0.49455000	-5.07572500	3.57223300
H	4.80198900	0.27791300	-3.45297200
H	5.97572300	1.44798600	1.50342300
H	4.13075800	-1.59370400	4.26847200
H	-5.19518700	2.21779500	-1.80442900
H	-1.78824800	2.87861400	-1.20244400
H	0.42543800	5.20953600	-5.42192700
H	-5.85405800	-2.71351800	4.95592000
H	5.12929400	5.80914700	-0.97308900
H	0.43005600	4.07416900	2.46784700
H	-2.66360900	-2.02354400	6.57490700
C	0.42674500	-0.13729200	0.98196200
C	1.39784100	0.03284500	-1.20089000
C	-0.47995100	0.84994200	0.60002500
C	0.42506200	0.97779000	-1.49738900
C	1.39299100	-0.54193400	0.06601100
C	2.46390500	-1.52825700	0.43892200
N	-0.49268300	1.39309000	-0.61725200
N	2.87812200	-3.60701900	1.59266700
N	2.01793700	-2.65715000	1.03102400
O	3.64789500	-1.31837700	0.22800600
H	0.41617700	-0.52581100	1.99486900
H	2.13963700	-0.25060800	-1.93887800
H	-1.20602700	1.23622600	1.30831400
H	0.37153600	1.41506100	-2.48971900
H	3.44435400	-4.01943300	0.85658700
H	3.49072800	-3.12475300	2.25422000
H	1.02913000	-2.84016500	1.12187100

ISO···β-CD complex in configuration F

Cartesian atomic coordinates:

C	-3.06116800	0.78817300	3.04901900
C	0.54377300	-2.12830500	4.38628900
C	4.02272900	-3.86032300	1.58485800
C	-4.12453600	3.82523100	-0.48367700
C	4.99016900	-2.01800100	-2.71972500
C	2.89035200	1.75930800	-4.91911300
C	-1.14673300	4.37813200	-4.17940900
C	-3.05553700	2.27808700	3.35166600
C	0.73112500	-0.63124000	4.52458300
C	4.26601000	-2.86849200	2.70545000
C	-3.26753200	5.07998200	-0.51467900
C	5.94460900	-1.77737600	-1.55360100
C	4.11641500	2.01829900	-4.06649100
C	0.20718400	5.01060800	-3.90780400
C	-4.38716200	2.90890700	2.95070300
C	-0.42394700	-0.03611200	5.30717800
C	3.73315500	-3.41341600	4.02496300
C	-3.43069500	5.84030800	-1.82594500
C	6.34463500	-3.11239300	-0.93031500
C	5.21898900	1.07469600	-4.51246100
C	1.11115900	4.84682600	-5.11447700
C	-3.38102200	0.58875100	1.57028500
C	-0.82313500	-2.43395800	3.76696000
C	2.54958200	-4.28003500	1.53881200
C	-3.81301200	2.97532100	-1.71760800
C	3.80201400	-2.89435500	-2.33426700
C	2.46577300	0.30044200	-4.78180400
C	-0.95792700	2.91201500	-4.58471900
C	-4.71613000	2.53307500	1.50666700
C	-1.74204900	-0.40708200	4.63431200
C	2.25983300	-3.77904800	3.85190500
C	5.08389100	-3.92037500	-0.60997600
C	-3.21828300	4.88102100	-2.99615200
C	4.72721200	-0.37536200	-4.48359000
C	1.20264100	3.36692900	-5.50807700
C	-3.40097400	-0.87489000	1.15800200
C	-1.12457500	-3.92188100	3.75552500
C	2.30464100	-5.44900300	0.60224000
C	-4.61385300	1.68687800	-1.85985100
C	2.97532400	-3.32830500	-3.53206400
C	1.22121700	-0.03904500	-5.58568200

C	-2.24155000	2.19676500	-4.97958900
O	-1.81157400	0.21645100	3.37404000
O	1.56460600	-2.59433600	3.52689700
O	-3.75444800	3.16118400	0.71295000
O	4.37478500	-3.20883300	0.37922800
O	4.52183800	-0.74290100	-3.15093600
O	1.87823600	2.67464900	-4.50448200
O	-1.86930200	4.50203000	-2.96656500
O	-4.68325100	1.13333600	1.34788600
O	-1.86913200	-1.79865500	4.51184000
O	2.10645500	-4.72242300	2.82693100
O	4.27609200	-4.10663700	-1.74432500
O	-4.07104400	3.77348100	-2.87577000
O	3.55226800	-0.50139900	-5.24736500
O	-0.09139700	2.86036200	-5.72394100
O	-2.82158100	2.46052600	4.74195600
O	1.96078400	-0.39339900	5.18501900
O	5.65716600	-2.62459400	2.77968500
O	-3.62833200	5.87702500	0.59923300
O	7.10065500	-1.11789500	-2.00350600
O	4.59665800	3.33935800	-4.22917600
O	-0.00825300	6.37377900	-3.59317700
O	-4.36796400	4.29974100	3.14606100
O	-0.22039300	1.35319400	5.40390200
O	3.93622800	-2.49667500	5.07173800
O	-2.55699100	6.94106600	-1.87867200
O	7.13806100	-2.99099100	0.21730000
O	6.36230300	1.18229000	-3.69154700
O	2.37698400	5.38475500	-4.76151900
O	-4.01544400	-1.05080200	-0.10704900
O	-1.11009900	-4.45883800	5.06028400
O	3.04595200	-6.58759500	0.98859600
O	-3.81866300	0.65621300	-2.43117700
O	3.74190000	-4.09012100	-4.43915900
O	1.23567100	0.57307800	-6.86454100
O	-2.11407000	0.79956900	-4.82804900
H	-3.85840400	0.29589900	3.62415200
H	0.61643800	-2.60321200	5.37199400
H	4.65551500	-4.74136100	1.74398900
H	-5.18784900	4.09827800	-0.45629500
H	5.54255300	-2.50784600	-3.52969100
H	3.13732100	1.93779100	-5.97048400
H	-1.65539800	4.93204400	-4.98036100
H	-2.24576400	2.74383700	2.77573500
H	0.74876600	-0.18167500	3.52380500
H	3.73376500	-1.93802700	2.46625100

H	-2.21923800	4.76459700	-0.42415400
H	5.41821100	-1.17834200	-0.79638300
H	3.88046800	1.82211000	-3.01223400
H	0.66426300	4.49117100	-3.05537100
H	-5.16983300	2.50414400	3.60128100
H	-0.43886000	-0.48375100	6.31042000
H	4.28838600	-4.31898500	4.28720300
H	-4.45119800	6.23129200	-1.88216400
H	6.93687200	-3.66736100	-1.66429500
H	5.47461500	1.31617100	-5.55208300
H	0.67815700	5.40124800	-5.95525500
H	-2.65820500	1.12699300	0.94797100
H	-0.82352600	-2.05342600	2.73792500
H	1.95204400	-3.41811800	1.21855200
H	-2.75125200	2.70492700	-1.68435900
H	3.16926100	-2.35373500	-1.61953900
H	2.27404600	0.06721300	-3.72564400
H	-0.49516700	2.37513300	-3.74764100
H	-5.72509900	2.85427500	1.23294200
H	-2.57730300	-0.08448400	5.26268800
H	1.84512600	-4.23198300	4.75627600
H	5.34246200	-4.91960700	-0.25097500
H	-3.46614700	5.36074700	-3.94683800
H	5.46019400	-1.03300700	-4.95596500
H	1.72015700	3.25437300	-6.46483000
H	-2.37290700	-1.22846900	1.07692400
H	-3.90144900	-1.47523600	1.92191800
H	-0.35731900	-4.42630800	3.16151800
H	-2.09705800	-4.07987800	3.27738700
H	1.22994000	-5.66602400	0.58767900
H	2.62467600	-5.18401800	-0.40639900
H	-4.98103700	1.36293600	-0.88465800
H	-5.46821400	1.88257800	-2.51345500
H	2.10913100	-3.89828600	-3.17387100
H	2.62401700	-2.45014800	-4.07425700
H	1.18423600	-1.12614500	-5.70468000
H	0.33700200	0.27498700	-5.02451300
H	-3.07323400	2.57346200	-4.38393800
H	-2.44174000	2.42416400	-6.03212800
H	-3.01336600	3.38486700	4.94617000
H	2.02316900	0.55161900	5.36525400
H	5.82806600	-2.15206900	3.60240700
H	-3.19072400	6.73083100	0.49996700
H	6.83648400	-0.28702600	-2.42475700
H	3.86705900	3.94721900	-4.04598700
H	0.85423700	6.79384000	-3.50240000

H	-3.96096300	4.72997200	2.37453300
H	-1.05631000	1.82319500	5.26689800
H	3.32182500	-1.74916100	4.97896000
H	-1.70266900	6.65151600	-2.23847700
H	6.60377100	-2.64472200	0.94777000
H	6.58420200	2.11700000	-3.61863900
H	2.86049300	5.63389000	-5.55451800
H	-4.97162400	-1.09101600	0.03235400
H	-2.02687900	-4.52023500	5.35840800
H	2.79008900	-6.80419800	1.89101700
H	-3.70717600	-0.03479600	-1.76094800
H	4.11660400	-4.82580600	-3.94332400
H	0.45230100	1.12877100	-6.91747400
H	-2.65120800	0.54596100	-4.06482300
C	-5.99207800	-1.51775500	3.77308000
C	-5.23025700	-3.58093900	2.84351700
C	-6.56408100	-1.23560400	2.53570700
C	-5.84303700	-3.21004700	1.65228800
C	-5.27294700	-2.70148600	3.92122200
C	-4.44766100	-3.06013500	5.13037700
N	-6.47397900	-2.04546200	1.48091100
N	-3.31290800	-2.19824200	7.06128400
N	-4.29691900	-2.09897500	6.07113900
O	-3.91364200	-4.15213300	5.22102200
H	-6.11958800	-0.80364900	4.57827500
H	-4.69954000	-4.51970800	2.93845900
H	-7.09833500	-0.30449700	2.38195900
H	-5.79791800	-3.86155900	0.78586500
H	-2.45716400	-2.52286100	6.60915200
H	-3.59988300	-2.90434400	7.73251900
H	-4.62417900	-1.16193800	5.90273500

ISO···β-CD complex in configuration G

Cartesian atomic coordinates:

C	-2.80152200	-1.05394000	-3.95367500
C	-2.87847100	-3.48662300	0.28352100
C	-2.12042400	-2.92602600	4.96093300
C	-0.65681500	3.12415200	-4.60828500
C	0.89544400	0.47691000	6.24001300
C	3.05355500	3.57086500	3.21866900
C	2.64511200	4.47487900	-1.45002600
C	-3.62789000	0.21309300	-3.85301900
C	-4.13448200	-3.07973200	-0.46406600
C	-3.46087200	-2.45755100	4.41985400
C	-1.05612900	4.13524900	-3.54769500
C	-0.58967200	0.68950400	6.48511900
C	1.73612800	3.92250900	3.87918000
C	2.30050200	5.45864900	-0.33588800
C	-3.50053100	1.04668000	-5.11694900
C	-4.04570900	-3.51290100	-1.92129300
C	-4.15851800	-3.56630400	3.64901400
C	-0.11913600	5.32550700	-3.58065900
C	-1.17450000	-0.42430100	7.34599200
C	1.89721500	3.78517800	5.38950700
C	3.49433300	5.60575400	0.60112500
C	-1.36744200	-0.75611000	-4.39989000
C	-1.68599400	-2.83265600	-0.40971400
C	-1.28935900	-3.49142700	3.80776400
C	0.82379800	2.75953500	-4.49055800
C	1.12650100	-0.92148000	5.65523300
C	3.47265600	2.15690600	3.63689500
C	3.16593200	3.14460600	-0.91549100
C	-2.01725900	1.26945600	-5.43147600
C	-2.73191400	-3.01860500	-2.53830200
C	-3.21729200	-4.09201400	2.56773000
C	-0.83148300	-1.76215500	6.69786900
C	1.32157000	4.83801200	-3.43313000
C	2.34803200	2.36512400	5.72654700
C	3.93854100	4.21320100	1.06644100
C	-0.57895800	-2.01945200	-4.68671100
C	-0.34651400	-3.07899600	0.25533400
C	0.05743900	-4.08250700	4.18615100
C	1.33221700	1.93749000	-5.66114900
C	2.59700200	-1.26083700	5.48273400
C	4.84458000	1.77792500	3.11284600

C	3.74410800	2.26188600	-2.00955200
O	-2.81738400	-1.61774000	-2.65272600
O	-3.00374700	-3.06438500	1.62839900
O	-1.48031000	1.99253100	-4.36192300
O	-1.50385300	-1.75674700	5.46940600
O	1.31941100	1.48823200	5.34047300
O	2.89047400	3.62431900	1.81487800
O	1.45834900	4.21304600	-2.19533000
O	-1.38324800	0.02915400	-5.59059700
O	-1.62921700	-3.36083900	-1.74036800
O	-2.01438700	-4.51999800	3.13438400
O	0.55707300	-1.88452800	6.54064300
O	1.61834000	3.94406400	-4.48471000
O	3.54878100	2.06260300	5.06366000
O	4.24711700	3.37215800	-0.01249000
O	-4.96996500	-0.18270300	-3.62316800
O	-5.25103800	-3.66683500	0.18526400
O	-4.23756700	-1.99982100	5.51195700
O	-2.37040400	4.61062600	-3.75886400
O	-0.75761900	1.95116100	7.10983800
O	1.38413100	5.25057100	3.54223100
O	1.97026300	6.71198600	-0.88514700
O	-4.17041300	2.27648400	-4.86592800
O	-5.16490200	-3.06944300	-2.65141100
O	-5.38203300	-3.09248100	3.13455500
O	-0.39754600	6.23589500	-2.53830400
O	-2.56126100	-0.25088400	7.51329900
O	0.72068700	4.16659800	6.05583000
O	3.25166000	6.42602300	1.70940900
O	-1.32713300	-2.88187600	-5.51755800
O	-0.09391300	-4.43349400	0.55683000
O	1.00752100	-3.89574000	3.15031200
O	1.16834300	2.62789000	-6.88335700
O	2.81204700	-2.55431600	4.97576000
O	5.83986600	2.68160300	3.54833300
O	4.87615700	2.85025600	-2.60984300
H	-3.27192700	-1.73190700	-4.67521400
H	-2.73731600	-4.57259500	0.24264100
H	-2.26757600	-3.66656100	5.75802400
H	-0.86085000	3.53440400	-5.60448600
H	1.43643100	0.56271300	7.19181200
H	3.81391800	4.29184500	3.54010700
H	3.40744700	4.91827700	-2.10137500
H	-3.25677100	0.80644700	-3.00880300
H	-4.21917600	-1.98457600	-0.42277500
H	-3.26656800	-1.62768900	3.72760800

H	-0.97421400	3.65567900	-2.56385200
H	-1.09741300	0.68692400	5.51248600
H	0.95933200	3.23062300	3.52930400
H	1.45664800	5.04802000	0.23597900
H	-3.96546400	0.50904100	-5.94993000
H	-4.06052300	-4.60679200	-1.95905900
H	-4.36579800	-4.39701800	4.33413400
H	-0.20787000	5.82352100	-4.55465400
H	-0.71922600	-0.38452900	8.33986800
H	2.68644500	4.47303400	5.71182500
H	4.32041100	6.06350600	0.04740000
H	-0.86250200	-0.20124300	-3.59986600
H	-1.83708400	-1.74728800	-0.46022100
H	-1.11248400	-2.65480600	3.12242800
H	0.96301900	2.20411800	-3.55486600
H	0.62472100	-0.98342600	4.67937500
H	2.72933600	1.44359700	3.25840600
H	2.35255600	2.61968400	-0.40067000
H	-1.87558800	1.80518000	-6.37547500
H	-2.56097900	-3.47441500	-3.51795100
H	-3.65506000	-4.96502700	2.07784100
H	-1.15082200	-2.60498200	7.31691100
H	2.02213900	5.66939900	-3.53348100
H	2.55469400	2.26704900	6.79523800
H	4.84546800	4.27995900	1.67297000
H	0.36321000	-1.72824300	-5.16422800
H	-0.34656300	-2.50775400	-3.73026100
H	-0.31866400	-2.52190400	1.19326100
H	0.43049200	-2.67033200	-0.40213900
H	0.46683400	-3.59132100	5.06894700
H	-0.08036900	-5.14562900	4.41157900
H	2.38704800	1.69813600	-5.48262600
H	0.76370600	1.01000100	-5.74131100
H	3.05233900	-0.48698700	4.85268900
H	3.08661400	-1.22985000	6.46004100
H	5.07382400	0.75439500	3.43097200
H	4.83671100	1.81216200	2.02155100
H	3.99187100	1.28403200	-1.57888100
H	3.00241400	2.12500000	-2.79726800
H	-5.50439900	0.61083500	-3.51681700
H	-6.00895400	-3.58434900	-0.40603300
H	-5.12645100	-1.82778200	5.17971600
H	-2.95913900	3.84662600	-3.84131100
H	-1.67032900	2.00217100	7.41889200
H	0.71518100	5.54166700	4.17354700
H	1.08804900	6.65217200	-1.27730900

H	-4.47490600	2.65933700	-5.69306200
H	-5.09167600	-2.11191800	-2.79984500
H	-5.35626200	-3.10589300	2.16568400
H	-1.34958200	6.38440100	-2.52153100
H	-3.02599900	-0.65818400	6.76367800
H	0.16397000	3.39098700	6.23807300
H	2.48205200	6.09819300	2.19985900
H	-0.72433500	-3.29526300	-6.15150300
H	0.27296300	-4.85397300	-0.24401300
H	0.69027200	-4.31867500	2.33479400
H	1.66495000	3.45001500	-6.81300600
H	2.34013200	-2.69881500	4.14342500
H	5.83690300	2.67469600	4.51094200
H	5.48305700	3.07995500	-1.89762200
C	2.45383300	-4.31373500	-3.69187800
C	0.33333200	-5.25521700	-4.29568400
C	2.11344100	-4.54190900	-2.36199600
C	0.09028400	-5.44067800	-2.94231000
C	1.54035400	-4.67713800	-4.67626100
C	1.77596300	-4.40099500	-6.13234500
N	0.95904500	-5.09165700	-1.99107500
N	3.40729800	-4.36446600	-7.90158300
N	3.05035800	-4.54625800	-6.56173100
O	0.87148400	-4.05664800	-6.87730900
H	3.39113100	-3.82837400	-3.93647400
H	-0.40506100	-5.52958300	-5.03802000
H	2.79153900	-4.26010700	-1.56274300
H	-0.84578800	-5.86759500	-2.60070200
H	3.34744200	-3.37295500	-8.11649000
H	2.71382800	-4.83212900	-8.48070100
H	3.73714000	-5.01145200	-5.98794100

ISO···β-CD complex in configuration H

Cartesian atomic coordinates:

C	-2.69684800	1.50596100	6.06179200
C	1.56482700	2.78916900	4.24500900
C	4.07117400	-0.05102500	1.32367600
C	-5.51667400	-2.42779600	5.79041100
C	2.55223300	-4.40559900	-0.02484600
C	-1.68938400	-6.66650200	0.89243200
C	-6.04329400	-6.11702100	2.93510000
C	-3.96419300	1.66760200	5.23526800
C	0.31116700	3.36252900	3.59620300
C	3.23294500	1.07926600	0.75450800
C	-6.88266500	-2.45275700	5.11296800
C	2.41231400	-3.21725600	-0.95725600
C	-1.27555300	-6.22183100	-0.49645000
C	-5.88712400	-5.65932000	1.48814400
C	-5.13557800	1.14465200	6.04035100
C	-0.58305800	4.01364800	4.63316800
C	3.64183100	2.39424700	1.38728800
C	-7.56177300	-3.79906300	5.34226900
C	3.77017600	-2.56237400	-1.15287900
C	0.12877400	-6.73619000	-0.78971300
C	-5.27970800	-6.73675200	0.60364400
C	-2.52851800	0.03832200	6.44504300
C	1.18228700	1.88873900	5.41618100
C	4.01849400	-0.05760300	2.85297100
C	-4.65730000	-3.59687700	5.33809600
C	3.23484300	-3.99275500	1.28234300
C	-0.69189000	-6.10356700	1.90270200
C	-4.70399900	-6.69691100	3.43212900
C	-4.87839900	-0.27116500	6.54565200
C	-0.86629100	3.03946200	5.77262300
C	3.54424600	2.27763700	2.90719500
C	4.37012000	-2.19721300	0.20984800
C	-6.59619000	-4.93884200	4.99922500
C	1.08883800	-6.29936300	0.32171900
C	-3.97954900	-7.24367900	1.20594100
C	-1.27053000	-0.25912700	7.23764700
C	2.34774900	1.40662600	6.25640600
C	5.00860300	-1.03536100	3.45811900
C	-3.32796700	-3.69639300	6.05990100
C	3.56414600	-5.18162400	2.16646900
C	-0.99382800	-6.48896200	3.33258700

C	-4.69782400	-7.47296900	4.74759800
O	-1.61453400	1.97067700	5.27187000
O	2.21278400	2.02614000	3.23450800
O	-4.90058400	-1.18050200	5.48552500
O	3.52378800	-1.25623300	0.80398700
O	1.24991600	-4.89787300	0.22018600
O	-3.00895800	-6.22299200	1.14772000
O	-6.43130700	-4.92563100	3.61124000
O	-3.65774800	-0.33343200	7.23866700
O	0.34940000	2.60730300	6.32225900
O	4.38072300	1.23471000	3.35004000
O	4.48397000	-3.35500200	0.99416400
O	-5.36314900	-4.79550500	5.65858700
O	0.60051900	-6.64173500	1.58786900
O	-4.20168800	-7.67485000	2.51168800
O	-4.24136900	3.01677900	4.93550500
O	0.61197100	4.34857600	2.63533400
O	3.40601400	1.21632200	-0.63995600
O	-7.70244300	-1.43201300	5.64671600
O	1.89577100	-3.69400200	-2.18610500
O	-2.21662600	-6.73393100	-1.42371200
O	-7.15661800	-5.28329700	0.99977700
O	-6.34219200	1.14568300	5.30565000
O	-1.81260300	4.41905300	4.06428300
O	2.80322800	3.44802300	0.95697000
O	-8.75472500	-3.94497100	4.61615400
O	3.57259100	-1.40820600	-1.96186400
O	0.57370900	-6.32957900	-2.06060800
O	-5.12461900	-6.24185300	-0.70805300
O	-1.06560400	0.61253600	8.32949700
O	1.90874500	0.51736600	7.25578200
O	6.33195400	-0.74795500	3.05415600
O	-2.52481900	-4.72895100	5.53703600
O	4.36774300	-6.12748500	1.49035200
O	-1.16375600	-7.88860200	3.49220200
O	-3.38802100	-7.50604400	5.28674600
H	-2.77102100	2.09449100	6.98290000
H	2.21335400	3.60754700	4.58433300
H	5.11144100	0.06458100	0.99873100
H	-5.65898200	-2.52512500	6.87325200
H	3.16021400	-5.17051500	-0.52316500
H	-1.64618600	-7.75945400	0.95790400
H	-6.84614200	-6.86422200	3.00921400
H	-3.86163200	1.07606800	4.31469600
H	-0.23711400	2.52705900	3.13987500
H	2.17956500	0.88752800	0.99560700

H	-6.73609500	-2.30148200	4.03467700
H	1.72564900	-2.48840800	-0.51013400
H	-1.27630200	-5.12340900	-0.53357600
H	-5.20876300	-4.79452200	1.49527900
H	-5.23684900	1.78496600	6.92804400
H	-0.05823300	4.87902200	5.05681900
H	4.68529400	2.60957400	1.12590900
H	-7.81325500	-3.87643300	6.40484900
H	4.43484100	-3.27347900	-1.65471600
H	0.09700200	-7.83004500	-0.80545900
H	-5.97168300	-7.58646300	0.57121400
H	-2.50823400	-0.57055400	5.53115500
H	0.63763500	1.02354400	5.01902700
H	3.00149100	-0.30951400	3.17715300
H	-4.47912000	-3.54527200	4.25665500
H	2.57992700	-3.30088700	1.82529800
H	-0.66071600	-5.00964600	1.83378000
H	-3.99756400	-5.86191000	3.51022600
H	-5.65171400	-0.52665600	7.26973200
H	-1.40182500	3.54051400	6.58182400
H	3.90535900	3.18749000	3.39304800
H	5.38448600	-1.80005500	0.11080400
H	-7.02155500	-5.89006000	5.33309400
H	2.05665800	-6.79808500	0.22321400
H	-3.62776500	-8.11951600	0.65496800
H	-1.31517900	-1.30415000	7.56609200
H	-0.40998300	-0.15674800	6.57537000
H	2.85255200	2.28041000	6.69133600
H	3.06299600	0.87226100	5.62760700
H	4.91108700	-1.01132600	4.54887300
H	4.78323300	-2.04341100	3.10597900
H	-2.78088500	-2.76137400	5.93136400
H	-3.51914200	-3.83696400	7.13158500
H	4.05877200	-4.81731600	3.07349500
H	2.64235800	-5.69011600	2.45231100
H	-0.19831700	-6.11212300	3.98277600
H	-1.92114900	-6.01440200	3.64425300
H	-5.31895600	-7.01956000	5.51027100
H	-5.06515400	-8.48421200	4.54176000
H	-3.47292900	3.41385500	4.49487900
H	1.24120200	3.98721200	1.99092100
H	3.21073900	0.36565600	-1.06024200
H	-7.36613400	-0.57079400	5.35481900
H	1.78049100	-2.94416900	-2.77877400
H	-1.83179800	-6.65708100	-2.30482100
H	-7.04749200	-5.09535700	0.06073200

H	-6.41217200	1.99803600	4.86080500
H	-1.60890600	4.90075100	3.25408900
H	2.75117900	3.39671100	-0.00497500
H	-8.51423000	-4.04254800	3.68575700
H	4.34699100	-1.25397400	-2.50972700
H	0.83442500	-5.39456100	-2.03152300
H	-4.18246000	-6.18989200	-0.92643500
H	-1.54963600	0.26395400	9.09435200
H	1.18611300	0.93518200	7.74740100
H	6.55110400	0.12842900	3.38633300
H	-2.93418300	-5.59393500	5.70482500
H	5.20989800	-5.70771300	1.28906800
H	-0.31515700	-8.32126000	3.36083800
H	-2.78732700	-7.93942100	4.66024300
C	-4.53577200	-2.41152400	9.56948000
C	-5.34038200	-0.21717500	10.04777200
C	-3.25743100	-1.94865700	9.85904600
C	-4.02644300	0.14599500	10.31368700
C	-5.60143100	-1.52183200	9.63148700
C	-6.93564300	-2.01958800	9.12003100
N	-2.99217100	-0.68854200	10.20367300
N	-9.11287700	-1.37486600	8.30177200
N	-7.87652600	-1.07731600	8.88611100
O	-7.11331500	-3.19856200	8.87159100
H	-4.71526400	-3.43613800	9.26738100
H	-6.11772500	0.52883700	10.16189300
H	-2.40468000	-2.61683400	9.78821700
H	-3.79197300	1.16380800	10.60912200
H	-9.47292400	-2.21036700	8.75379700
H	-8.93995700	-1.59525300	7.31887000
H	-7.72669500	-0.10618800	9.09954000

ISO···β-CD complex in configuration I

Cartesian atomic coordinates:

C	2.58069000	2.91460500	3.56330200
C	3.52496400	-1.58258400	2.14469700
C	1.17303600	-3.99490900	-1.31202500
C	-1.29582500	5.38241400	2.21086700
C	-2.88670000	-3.28313900	-3.92299600
C	-5.05239600	-0.14461100	-3.29203900
C	-4.47481100	4.16779400	-1.23730800
C	2.72380600	3.94805700	2.46198900
C	4.31434000	-0.41715600	1.57221900
C	2.54053200	-3.37595900	-1.55445100
C	-1.23722100	6.07598900	0.84991200
C	-0.87764600	-2.05629000	-4.47552900
C	-4.40838400	-0.41183600	-4.64062600
C	-3.86181300	3.91693500	-2.60554700
C	2.20485100	5.28425900	2.95843700
C	4.91308700	0.41912400	2.68576900
C	3.51190400	-3.86852900	-0.49835400
C	-2.60958800	6.58802500	0.42698100
C	-0.16126900	-3.40868600	-4.57628700
C	-4.66432200	-1.84772300	-5.07121500
C	-4.92989000	3.40805900	-3.56229700
C	1.14373300	2.87383600	4.08289600
C	2.50845500	-1.08620600	3.17249100
C	0.70055300	-3.74211500	0.11798100
C	-2.40510400	4.33616500	2.24970500
C	-2.51365900	-4.19868600	-2.75501900
C	-4.57995900	-1.17032600	-2.26681800
C	-5.22449000	2.92199300	-0.74904000
C	0.77516200	5.13211700	3.47059200
C	3.82420000	0.82920500	3.67550000
C	2.95310500	-3.59009600	0.89529400
C	-0.12958000	-4.24143000	-3.29565200
C	-3.66929800	5.49713200	0.61837400
C	-4.35865500	-2.88987800	-3.98232000
C	-5.61466300	2.17443300	-2.97671200
C	0.99070000	1.91485700	5.24525100
C	1.79102900	-2.22328200	3.87603800
C	-0.56617400	-4.50921100	0.45264900
C	-2.59048300	3.70860200	3.61728700
C	-3.51397700	-5.27358500	-2.36053300
C	-5.31058400	-1.03846900	-0.94025500

C	-6.02388800	3.17561000	0.51679700
O	2.92402600	1.65717200	2.99779600
O	2.85368800	-2.19853100	1.05546400
O	-0.03551800	4.75115300	2.39579600
O	0.24682700	-3.42046900	-2.22648200
O	-2.09800000	-2.09883400	-3.80020000
O	-4.66793700	1.15252300	-2.85664700
O	-3.42445200	4.49679700	-0.35312400
O	0.74069900	4.17498200	4.50257400
O	3.18268500	-0.32177600	4.17498700
O	1.69750100	-4.20165400	1.03582500
O	-1.31975100	-4.93322700	-3.04410100
O	-3.63864200	4.95323800	1.90503300
O	-4.89986600	-2.48216700	-2.73709900
O	-6.17324500	2.50092000	-1.73180800
O	4.06828400	4.14112000	2.08467900
O	5.38165200	-0.85301200	0.74946600
O	3.08229600	-3.74248400	-2.80472400
O	-0.34011900	7.15341600	0.88237800
O	-1.01987900	-1.52163200	-5.76870900
O	-4.95709700	0.39927200	-5.66287800
O	-3.22837500	5.06805500	-3.14701700
O	2.21824800	6.25397200	1.93066000
O	5.51960200	1.59080200	2.17153600
O	4.76310400	-3.21659600	-0.60863800
O	-2.63632800	7.03278000	-0.90885400
O	1.19276100	-3.15706000	-4.92116700
O	-3.90865200	-2.15667700	-6.23746700
O	-4.37813800	3.07733100	-4.82021500
O	2.05338900	2.12669900	6.15723600
O	2.67724600	-3.17693100	4.41467700
O	-0.45322800	-5.88648800	0.15100800
O	-3.44488800	2.58011400	3.57764500
O	-3.11982500	-5.89024200	-1.15784400
O	-6.69800100	-1.23983900	-1.08864700
O	-6.95028200	4.22917900	0.33091300
H	3.25775300	3.16216900	4.38656200
H	4.21139300	-2.29162700	2.62292100
H	1.22827700	-5.07763000	-1.46794700
H	-1.46722000	6.13421600	2.99245200
H	-2.67776300	-3.79226000	-4.86981900
H	-6.14388500	-0.20906100	-3.37115900
H	-5.18892500	5.00043200	-1.30573700
H	2.12025300	3.63113700	1.60087900
H	3.62793300	0.21410000	0.99306800
H	2.45162200	-2.28462700	-1.47264700

H	-0.91408900	5.31493000	0.12167300
H	-0.23703500	-1.37386500	-3.91544300
H	-3.33234100	-0.23837600	-4.55213800
H	-3.07545200	3.16246100	-2.51764600
H	2.82895700	5.61286500	3.79846400
H	5.65196900	-0.18459900	3.22357300
H	3.64288000	-4.95189900	-0.60897500
H	-2.87043500	7.44604300	1.05182400
H	-0.62658900	-4.02054200	-5.35694300
H	-5.73429500	-1.92237600	-5.30350700
H	-5.70177300	4.18137100	-3.68354900
H	0.48952000	2.53604700	3.26793200
H	1.76784100	-0.45491500	2.66527100
H	0.52845600	-2.66882700	0.26598000
H	-2.16626800	3.55239600	1.51621800
H	-2.34557100	-3.54736700	-1.89110900
H	-3.49941200	-1.06631300	-2.11455500
H	-4.48879300	2.12827100	-0.57110700
H	0.42233600	6.06762700	3.91105800
H	4.24370000	1.34057200	4.54418600
H	3.58739000	-4.02054600	1.67330600
H	0.61523700	-5.02759700	-3.44994700
H	-4.67426200	5.90646600	0.48939600
H	-4.90579600	-3.80340700	-4.23375000
H	-6.44560800	1.85994400	-3.61231500
H	0.01744900	2.09266300	5.71597900
H	1.02416300	0.89298600	4.85171100
H	1.17325800	-2.75366900	3.14840100
H	1.13910100	-1.79873800	4.64922200
H	-0.80115800	-4.35632700	1.51130300
H	-1.39695100	-4.13310700	-0.14182100
H	-1.61144300	3.45972400	4.03927000
H	-3.07393400	4.43425200	4.27424400
H	-4.48806800	-4.82322400	-2.17283400
H	-3.60344700	-6.00221500	-3.17792600
H	-4.88130800	-1.75247100	-0.22754900
H	-5.17573100	-0.02792900	-0.55314500
H	-6.53320600	2.24650100	0.79491000
H	-5.35940400	3.46790700	1.33085700
H	4.49512800	3.27742600	1.96951100
H	5.03464800	-1.51367700	0.12207500
H	2.53661500	-3.38197600	-3.52075400
H	0.54421600	6.81939400	1.09805800
H	-1.87932700	-1.81305200	-6.11376800
H	-4.65794400	1.31210500	-5.52894100
H	-3.81819100	5.82626900	-3.05159900

H	3.08501400	6.21603200	1.50961500
H	6.07930900	1.32441900	1.43285000
H	5.07885000	-3.30959100	-1.51707400
H	-2.32221200	6.30588500	-1.46273900
H	1.20020700	-2.58371100	-5.69659100
H	-4.17302300	-1.50808900	-6.90186500
H	-3.72061300	3.74772700	-5.04089500
H	1.98075700	1.49140800	6.87251700
H	3.30420000	-2.74201100	5.00736400
H	0.20120400	-6.27381000	0.74030400
H	-3.01438400	1.88957600	3.06763900
H	-2.20082900	-6.18210000	-1.22601800
H	-6.81892800	-2.12005900	-1.45915300
H	-7.58635500	3.94134200	-0.33120100
C	5.86243900	-4.74790000	3.05093000
C	7.01247600	-2.65650800	3.19531500
C	5.32126400	-4.47803300	4.30202300
C	6.43650800	-2.49496000	4.45192000
C	6.70209500	-3.80304800	2.47091000
C	7.20965900	-4.06561300	1.08189200
N	5.58844300	-3.36778100	4.99426400
N	7.71358900	-3.10096700	-1.02116500
N	7.53669900	-2.94871900	0.37113000
O	7.29406100	-5.19072500	0.62918800
H	5.63224300	-5.66237900	2.51777300
H	7.68819500	-1.90570900	2.80316800
H	4.62435000	-5.17049100	4.76112700
H	6.65974800	-1.61434800	5.04726800
H	8.70148400	-2.97890300	-1.22518300
H	7.50202900	-4.07862100	-1.22326500
H	7.02548500	-2.10577200	0.61721400

ISO···β-CD complex in configuration J

Cartesian atomic coordinates:

C	4.48222000	-1.50227000	0.82588600
C	4.39498600	0.73713900	-3.41792500
C	0.82077200	2.84528800	-5.74046900
C	1.03925000	-1.74807600	4.25905500
C	-3.72609500	2.93530100	-4.55989100
C	-5.10690800	1.15329300	-0.27945500
C	-3.46333300	-0.27788400	3.97461300
C	4.65261400	-0.72323200	2.11800900
C	5.18334500	1.39721900	-2.29663600
C	1.80632100	3.79706500	-5.06576700
C	0.71638400	-0.47347700	5.02096700
C	-2.94170500	4.21919800	-4.34032000
C	-5.46344000	2.58578100	-0.67066900
C	-3.73449400	1.21315700	3.82833500
C	4.44691600	-1.63057600	3.32326800
C	6.18015500	0.41005900	-1.69880700
C	3.22875000	3.44501800	-5.48819600
C	-0.47053300	-0.66938200	5.96083100
C	-2.27685900	4.68527200	-5.62056400
C	-6.10650900	2.59184500	-2.05459800
C	-5.14812700	1.44758400	3.32744000
C	3.09394800	-2.15022800	0.82195000
C	3.69498400	-0.50267800	-2.84821800
C	1.16684000	1.39456700	-5.41997900
C	-0.21648100	-2.25477300	3.54808600
C	-2.89124900	1.88185900	-5.30211600
C	-4.15794400	0.57030800	-1.31980500
C	-3.77930200	-0.98057200	2.64718400
C	3.11697000	-2.37534100	3.19094800
C	5.45993600	-0.87746700	-1.30665100
C	3.46420300	1.94299000	-5.29768100
C	-1.45680000	3.53231900	-6.20208800
C	-1.63060300	-1.31445700	5.21026400
C	-5.24078700	1.78112900	-3.03286100
C	-5.37464000	0.66756300	2.03124000
C	2.75941300	-2.98931700	-0.40800900
C	2.80304700	-1.29102700	-3.81240700
C	0.23824400	0.34166100	-6.03104300
C	-0.06462300	-3.52994500	2.70773700
C	-3.71730100	0.68640200	-5.75390600
C	-3.63307300	-0.81813600	-1.03853900

C	-3.65714700	-2.49817500	2.64120100
O	4.62693100	-0.56330000	-0.22279000
O	3.48231400	1.69648000	-3.91107100
O	2.08710300	-1.43000400	3.36541100
O	-0.47112300	3.19942000	-5.26937200
O	-4.06462600	2.51270900	-3.24973300
O	-4.54495600	1.18061500	1.02898000
O	-2.08523300	-0.36216300	4.29291600
O	3.00965600	-3.01938700	1.95592900
O	4.72584500	-1.38855300	-2.38693800
O	2.48563900	1.16415000	-5.93200700
O	-2.31543500	2.46020300	-6.47506400
O	-1.19802000	-2.49261300	4.57310500
O	-4.90670000	0.50719100	-2.53703300
O	-5.12250400	-0.69178500	2.26311600
O	5.94428000	-0.14551400	2.11508400
O	5.84328300	2.52795200	-2.82852400
O	1.52090900	5.12485400	-5.43747800
O	1.87475000	-0.10551500	5.75391700
O	-3.84871400	5.18618800	-3.83988100
O	-6.35423700	3.14024700	0.27003400
O	-3.51243600	1.81565500	5.08819900
O	4.52204500	-0.89716600	4.52483200
O	6.87753500	0.97757000	-0.61682200
O	4.21711400	4.18581900	-4.82764200
O	-0.84638500	0.54744200	6.56031900
O	-1.46809800	5.80736700	-5.29095200
O	-6.32200900	3.87501500	-2.57333900
O	-5.33763300	2.84437700	3.13819500
O	1.44294300	-2.75549300	-0.88010300
O	1.45150700	-1.33942200	-3.37991700
O	-0.54274800	-0.31733300	-5.04665600
O	-0.33847600	-3.28355100	1.33449700
O	-2.95396300	-0.29271000	-6.40925200
O	-4.65247200	-1.75398600	-0.72331700
O	-3.22670700	-2.98845900	1.38185000
H	5.25835000	-2.27601500	0.75484100
H	5.08267000	0.42478400	-4.21503900
H	0.87288600	2.98738600	-6.82818800
H	1.37430900	-2.50650000	4.97271600
H	-4.63703100	3.17302600	-5.12750000
H	-6.01065500	0.53389700	-0.27318700
H	-4.06955400	-0.68668900	4.79460300
H	3.88930900	0.06761800	2.13600100
H	4.47377000	1.70722100	-1.51707500
H	1.71692400	3.66927200	-3.97774800

H	0.46925300	0.30868300	4.29091900
H	-2.15724700	4.01425300	-3.60230800
H	-4.53425600	3.17231700	-0.71190700
H	-3.03156600	1.61256100	3.08566600
H	5.25217100	-2.37211800	3.35339100
H	6.91970600	0.16534800	-2.46810200
H	3.32029400	3.66492500	-6.55727900
H	-0.17207400	-1.35167500	6.76306500
H	-3.04829600	4.96290800	-6.34688900
H	-7.08823200	2.11524000	-1.97364400
H	-5.85198500	1.06369100	4.07377300
H	2.34241100	-1.35634700	0.90914500
H	3.07312200	-0.19469700	-1.99926700
H	1.16972200	1.25475600	-4.33243800
H	-0.58075600	-1.46781900	2.87892300
H	-2.09193700	1.54003100	-4.63555300
H	-3.29206700	1.22841200	-1.45654300
H	-3.08159700	-0.57530700	1.90553200
H	3.04725400	-3.16184400	3.94435700
H	6.17757500	-1.65356100	-1.02706900
H	4.42050500	1.65830000	-5.74392700
H	-1.00521600	3.81472600	-7.15838000
H	-2.42780200	-1.60834900	5.90013100
H	-5.78295600	1.62183100	-3.96780000
H	-6.42323400	0.73433100	1.73146000
H	3.42854300	-2.74359300	-1.23146200
H	2.90165300	-4.04352200	-0.14857600
H	3.21776500	-2.29940500	-3.90851700
H	2.79254200	-0.82522800	-4.79821900
H	-0.47182200	0.79039800	-6.72965700
H	0.85826100	-0.37432400	-6.58073200
H	0.95104600	-3.92009400	2.75879900
H	-0.73365700	-4.29363200	3.11331400
H	-4.22311500	0.26692800	-4.87599800
H	-4.47502200	1.02834100	-6.46581700
H	-3.05404700	-1.14953300	-1.90661900
H	-2.96644900	-0.78567700	-0.17695700
H	-2.91665200	-2.82909000	3.36496200
H	-4.62937000	-2.92095800	2.91546500
H	6.11578800	0.18729900	3.00370500
H	6.46304500	2.84379200	-2.16074200
H	0.63145800	5.32869800	-5.11818900
H	1.64705900	0.66192000	6.29147700
H	-3.35555000	5.99662600	-3.67563700
H	-5.86221600	3.25960300	1.09298100
H	-3.68422400	2.75878700	4.99445400

H	3.65446000	-0.50393600	4.71539300
H	6.40287400	0.78629200	0.20794800
H	4.48299000	3.71828400	-4.02258600
H	-1.47534200	0.99758700	5.97503800
H	-1.38698200	6.38432400	-6.05525100
H	-5.46642200	4.28793500	-2.76823400
H	-6.18774500	3.10485100	3.50153600
H	0.80575100	-2.97885400	-0.17882100
H	1.37513500	-1.84490300	-2.55076600
H	0.05494500	-0.78093600	-4.43388200
H	-1.30135600	-3.22553800	1.21157300
H	-2.20772900	-0.53522200	-5.83916300
H	-5.20166800	-1.88779600	-1.50205800
H	-3.88287100	-2.76913000	0.69932300
C	3.23111900	-5.54217200	5.33895800
C	3.20082100	-3.51394400	6.60772900
C	4.59674700	-5.34984100	5.17058900
C	4.57006700	-3.41022100	6.36827300
C	2.51493000	-4.60191300	6.07391400
C	1.01830000	-4.75077300	6.11631700
N	5.26224800	-4.30303800	5.66357400
N	-1.02862700	-4.04487400	7.16373800
N	0.37033500	-4.07694500	7.10071800
O	0.42244700	-5.39377500	5.27309300
H	2.71625000	-6.38128400	4.88867700
H	2.69855700	-2.70633700	7.12915400
H	5.18290100	-6.06263400	4.59958300
H	5.12138100	-2.55092500	6.73190100
H	-1.35709300	-4.97194800	7.42589900
H	-1.34841500	-3.88288400	6.20901400
H	0.86146600	-3.59868400	7.83820300

Table S2. Calculated μ and ω parameters for β-CD, ISO, and ISO···β-CD complex in configurations A–E.

System	Configuration	μ (eV)	ω (eV)
**β-CD**		–4.80	2.84
**ISO**		–5.06	3.15
	**A**	–4.86	3.22
	**B**	–5.17	3.64
**ISO···β-CD**	**C**	–4.71	3.13
	**D**	–5.15	3.67
	**E**	–5.32	3.73
